# A photosynthetic bacterial inoculant exerts beneficial effects on the yield and quality of tomato and affects bacterial community structure in an organic field

**DOI:** 10.3389/fmicb.2022.959080

**Published:** 2022-08-29

**Authors:** Sook-Kuan Lee, Ming-Shu Chiang, Zeng-Yei Hseu, Chih-Horng Kuo, Chi-Te Liu

**Affiliations:** ^1^Institute of Biotechnology, National Taiwan University, Taipei, Taiwan; ^2^Department of Agronomy, National Taiwan University, Taipei, Taiwan; ^3^Department of Agricultural Chemistry, National Taiwan University, Taipei, Taiwan; ^4^Institute of Plant and Microbial Biology, Academia Sinica, Taipei, Taiwan; ^5^Agricultural Biotechnology Research Center, Academia Sinica, Taipei, Taiwan

**Keywords:** PGPR, biostimulant, microbiota, soil health, sustainability

## Abstract

Plant growth-promoting rhizobacteria (PGPR) are microorganisms that promote plant health and play a critical role in sustainable agriculture. As a PGPR, *Rhodopseudomonas palustris* strain PS3, when applied as a microbial inoculant, exhibited beneficial effects on a variety of crops. In this study, we investigated the effects of PS3 on tomato growth, soil properties, and soil microbiota composition in an organic field. The results demonstrated that PS3 inoculation significantly improved the yield of marketable tomato fruit (37%) and the postharvest quality (e.g., sweetness, taste, vitamin C, total phenolic compounds, and lycopene). Additionally, soil nutrient availability (35–56%) and enzymatic activities (13–62%) also increased. We detected that approximately 10^7^ CFU/g soil of *R. palustris* survived in the PS3-treated soil after harvest. Furthermore, several bacterial genera known to be associated with nutrient cycling (e.g., *Dyella, Novosphingobium, Luteimonas, Haliangium,* and *Thermomonas*) had higher relative abundances (log2 fold change >2.0). To validate the results of the field experiment, we further conducted pot experiments with field-collected soil using two different tomato cultivars and obtained consistent results. Notably, the relative abundance of putative PGPRs in the genus *Haliangium* increased with PS3 inoculation in both cultivars (1.5 and 34.2%, respectively), suggesting that this genus may have synergistic interactions with PS3. Taken together, we further demonstrated the value of PS3 in sustainable agriculture and provided novel knowledge regarding the effects of this PGPR on soil microbiota composition.

## Introduction

Plant growth-promoting rhizobacteria (PGPRs) are rhizosphere microorganisms that colonize plant roots and improve yield (Kloepper et al., 1980). PGPRs can enhance plant growth by multiple mechanisms, such as increasing the uptake of essential nutrients (i.e., phosphate solubilization, siderophore production, and biological nitrogen fixation), mitigating abiotic stress (i.e., production of 1-aminocyclopropane-1-carboxylate deaminase and ACC), enhancing resistance to plant diseases (i.e., PGPR-induced systemic resistance and ISR), modifying phytohormonal content, and altering the rhizosphere microbiota (Spaepen and Vanderleyden, 2011; Glick, 2014; Hakim et al., 2021; Mohanty et al., 2021). Common PGPR include those in the genera *Pseudomonas*, *Azospirillum*, *Azotobacter*, and *Bacillus,* which play an essential role in organic farming and agricultural sustainability as effective microbial inoculants (Oancea et al., 2017; Castiglione et al., 2021; Hamid et al., 2021).

*Rhodopseudomonas palustris* is a photosynthetic bacterium that can be applied as a PGPR inoculant to improve crop yield and quality, as well as alleviate biotic and abiotic stress for crops (Sakarika et al., 2020; Lee et al., 2021a). The deduced plant growth-promoting mechanisms of the plant-associated beneficial bacterium *R. palustris* can be summarized as facilitating nutrient acquisition in the soil or rhizosphere (i.e., biofertilization), producing phytohormones (i.e., biostimulation), and inducing the immune system in plants (i.e., biocontrol; Lee et al., 2021a).

*Rhodopseudomonas palustris* strain PS3 was isolated from rice paddy soil in Taiwan (Wong et al., 2014). This strain could utilize different carbohydrate sources, produce versatile extracellular hydrolytic enzymes, fix nitrogen, and produce indole-3-acetic acid (IAA) in a free-living state (Wong et al., 2014). When PS3 was inoculated in soil treated with chemical fertilizer or in a hydroponic system, it enhanced fertilizer efficiency and exerted beneficial effects on leafy crops (Wong et al., 2014; Hsu et al., 2015, 2021). In this study, we aimed to further understand the effects of PS3 on fruit crops, soil health, and the microbial community in an organic field. To achieve this goal, we conducted a field trial on tomato production. Furthermore, to establish the potential environmental impact on the soil microbial community, we performed a microbiota survey based on high-throughput sequencing of 16S rDNA gene amplicons throughout the flowering and fruit set periods.

## Materials and methods

### Preparation of microbial inoculant

*Rhodopseudomonas palustris* strain PS3 (international culture collection catalog numbers: BCRC 910564; DSM 29314) was cultivated in modified van Niel medium supplemented with CH_3_COONa (2 g/L) and peptone (5 g/L; i.e., modified PNSB medium) as described previously (Lo et al., 2020). Fifty milliliters of the precultured broth was inoculated into a 2,000 ml Erlenmeyer flask containing 600 ml of fresh modified PNSB broth and cultivated in the dark at 37°C and 200 rpm for 22 h. After culturing, 600 ml of the inoculum was transferred into 12 L of fresh modified PNSB broth to produce large-scale cultures until the cell density reached approximately OD_600_ = 2.0 and adjusted to a concentration of OD_600_ = 1.0 [approximately 1 × 10^9^ colony forming units, (CFU)/ml] for further experiments.

### Experimental field

This study was carried out on a Town-South organic farm (24.29°N, 120.40°E) located in Tonghsiao Township, Miaoli County, Taiwan (Supplementary Figure S1A). The experiment was conducted in a greenhouse; the soil moisture, temperature, illumination, and electroconductivity (EC) were monitored and recorded with a VegTrug Soil Tester (Xiaomi Co., Ltd.). During the planting period, the average soil temperature and moisture were 23.7°C and 24.82%, respectively. Moreover, the sunlight intensity was approximately 5,000 lux inside the greenhouse, and the EC was 235.5 μS/cm for the experimental farmland soil (Supplementary Figure S1B).

### Experimental design for the field trial

The commercial tomato (*Lycopersicon esculentum Mill*.) hybrid cv. “Yu Nu” was used as the plant material, which was grafted onto an eggplant rootstock to make it more disease-resistant (Liu et al., 2009). We grew the tomato plants in 0.8 m × 8.0 m plots with a row spacing of 60 cm and a plant spacing of 25 cm, and we cultivated approximately 40 plants per furrow at the vegetative stage. In each row, 10 of the plants were labeled and used for investigating the agricultural traits and soil biochemical process (Supplementary Figure S2A). Due to limitations of the commercial farm site’s irrigation and planting systems, the PS3 inoculant was applied to one of two neighboring plots in a two-block design. The soil properties of the control group (i.e., OF+M, applying plant-based organic fertilizer on a regular basis depending on the routine of the farm, supplemented with modified PNSB medium) and the experimental group (i.e., OF+PS3, applying plant-based organic fertilizer, supplemented with PS3 inoculant) were similar, suggesting that the initial environments resembled each other (Supplementary Table S1). The timeline for sampling and inoculation is illustrated in Supplementary Figure S2B. Two hundred milliliters of modified PNSB broth or the PS3 inoculant (fermented broth; OD_600_ = 1.0, equivalent to 10^9^ CFU/ml) was poured into the rhizosphere of individual tomato plants during their vegetative, flowering, and ripening stages. A drip irrigation system was used on this farm, and the plant-based organic fertilizer 426 (Fwusow industry Co., Ltd., Taiwan) was applied as base fertilizer, and a top dressing fertilizer was applied at the stage of fruit ripening. We observed differences in the inflorescence and fruiting branches of tomato plants during the growing season. We periodically harvested the 10 labeled red ripe tomatoes from the beginning until the end of the production period. For each treatment, we collected the soil samples from each plant associated with roots and packed them into zipper bags stored on ice-cold packs before analysis. The soil samples were air-dried to measure soil physicochemical properties (plant-available nutrients) or stored at −20°C for soil enzyme activities. Five soil samples were collected from the respective rhizospheres of each plant and treatment for DNA extraction (Supplementary Figure S2B).

### Analysis of yield parameters of tomato fruit

The numbers of inflorescence and fruiting branches of the tomato plants were investigated during the whole cultivation period. We harvested tomato fruit from individual labeled plants (*n* = 10) at the ripening stage to determine the agronomic traits. The fresh weights of 50 fruits per plant were determined, and the fruits were dehydrated in an oven at 65°C (for at least 72 h) to constant weights to determine the dry weights. The marketable yield was determined by summing the weight of all the tomato fruits.

### Analysis of fruit quality

The total soluble solid (TSS or Brix) content and total acidity were measured by a portable Brix-acidity meter (PAL-BX|ACID3 Master Kit, ATAGO Co., Ltd., Tokyo, Japan). The sugar/acid ratio (°Brix ÷ % Acid) was calculated by dividing the Brix degree with the citric acid percentage. The lycopene levels in the fresh fruit were determined using the method described by (Giovannetti et al., 2012) with some modifications. The vitamin C levels in fresh fruit were extracted according to the application note provided by Merck (Merck, Germany). The vitamin C test strip (Merck, Germany) was stained with the sample solution and measured by a reflectometer (Merck KGaA, Darmstadt, Germany). The calculation formula was as follows: obtained value (milligrams/liter) × 4. The total phenolic levels of the extracts were determined using Folin and Ciocalteu reagent following the method described by (Chandra et al., 2014) with some modifications. The phenolic content was calculated as gallic acid equivalents (GAE)/g of dry plant material. All determinations were carried out in 20 replicates per plant.

### Root analysis

Plant growth-promoting rhizobacteria can alter root system development and growth through the production of phytohormones, secondary metabolites, and enzymes (Grover et al., 2021). To investigate the effects of PS3 on roots, we analyzed the morphology and histology of the roots. After the harvesting season ended, individual roots of the labeled tomato plants were sampled and weighed. Five adventitious roots were collected from each plant. According to the distance between the adventitious roots and the taproots, each adventitious root was cut and divided into three parts: where T represents the portion in closest proximity to the taproots, M represents the portion midway between the taproots and soil, and B represents the portion far away from the taproots. Slice preparation and toluidine blue staining were performed as described by Li et al. (2017) with some modification. The slices were photographed using a light microscope (OLYMPUS BX51, Japan). ImageJ was used for area quantification and analysis.

### Soil physicochemical analysis

The soil pH and soil electrical conductivity (EC) were determined by a 1:5 soil/deionized water suspension with a pH meter (TOA-DKK, HM-41X, Japan) and EC meter (Field scout, FSEC-20, United States). The organic carbon (SOC) and organic matter (SOM) concentrations (%) in the soil were measured by the Walkley black method (Nelson and Sommers, 1996). In addition, the potassium permanganate test was used to determine the soil active carbon (SAC) content (Weil et al., 2003). Moreover, total nitrogen was analyzed by Kjeldahl digestion (Bremner, 1996). The soil extract (20 ml) was distilled after the addition of MgO and Devarda’s alloy to quantify the levels of ammonia (NH_4_^+^-N) and nitrate (NO_3_-N), respectively (Keeney and Nelson, 1983).

We also determined the exchangeable potassium (Ex.K), exchangeable calcium (Ex.Ca), and exchangeable magnesium (Ex.Mg) as described by Mehlich (1984). Phosphorus (P) availability was determined with the Bray P method (Olsen and Sommers, 1983). The levels of K, Ca, Mg, and P were quantified by atomic absorption spectrometry (AAS; PE Analyst 200, PerkinElmer, Inc., United States).

### Soil extracellular enzyme activities and biological properties

To determine the function and nutrient cycling capacity of the soil microbiota, we assessed the activities of the extracellular enzymes isolated from the soil as described by Yang et al. (2019). Dehydrogenase activities in the soil samples were determined following the method described by Casida et al. (1964) and quantified by a spectrophotometer (Ultrospec 2,100 pro, Cambridge Scientific Co., Ltd., America) at 485 nm. The phosphatase activities were determined according to the method described by Tabatabai and Bremner (1969) and was quantified the absorbance at 410 nm. Urease activity was measured using a colorimetric method (Bremner and Douglas, 1971) and measured at a wavelength of 578 nm using a spectrophotometer.

### DNA extraction and Illumina sequencing

Five soil samples were collected from the respective rhizospheres of each plant and treatment for DNA extraction. For each sample, 0.6 g of the soil was used for total DNA extraction with the soil FAST DNA spin kit (MP Biomedicals, United States) according to the manufacturer’s instructions. The concentration of DNA was determined using a fluorometer (Nanodrop ND-1000, J and H Technology Co., Ltd), and the purity of the DNA was monitored using 1% agarose gels. The bacterial V3-V4 regions of 16S rDNA were amplified by PCR using 341F (5′CCTACGGGNGGCWGCAG-3′) and 806R (5′ GACTACHVGGGTATCTAATCC-3′). Sequencing libraries were generated using the TruSeq Nano DNA Library Prep Kit (Illumina, United States) following the manufacturer’s recommendations, and index barcodes were added. The library quality was assessed by a Qubit 2.0 Fluorometer (Thermo Scientific) and an Agilent Bioanalyzer 2100 system. The library was finally sequenced using an Illumina MiSeq platform to generate 300 bp paired-end reads by Genomics BioSci and Tech Co., Ltd. (New Taipei, Taiwan).

### Classification and taxonomic assignment of OTUs

Sequences of primers were removed from the demultiplexed sequences using Cutadapt 1.8.1 (Martin, 2011). Data for 16S rDNA were analyzed with QIIME2 version 2020.6 (Bolyen et al., 2019). The paired-end reads were merged using the command “qiime vsearch join-pairs.” Then, joined sequences were filtered by quality (Phred score * 20) and dereplicated with VSEARCH. UCHIME was used to eliminate singletons or chimeras, resulting in 68% of reads remaining (68% was a count from chimera removing sequences divided by OTU clustering sequences × 100%; Supplementary Table S2). Before downstream analysis, we clustered representative sequences into operational taxonomic units (OTUs) with a threshold of 99% sequence identity by using the q2-vsearch plugin. Subsequently, taxonomy was assigned by the “classify-sklearn” function of the “feature classifier” plugin with a Naïve Bayes classifier trained on SILVA 132, using 99% OTUs full-length sequence of 16S rRNA genes (Quast et al., 2013; Yilmaz et al., 2014). Across the 10 samples, qualified sequences were detected, and representative OTUs were aligned with MAFFT using the “alignment” plugin in QIIME2. Variable positions were masked using the “mask” function in QIIME2 (Katoh and Standley, 2013). The phylogenetic tree was built using the “Fasttree” function within the “phylogeny” plugin. (Price et al., 2010).

### Quantitative PCR

Real-time quantitative PCR (qPCR) was performed in five biological repeats and three technical repeats with a LightCycler® 480 Real-Time PCR System (Roche Applied Science). Each qPCR consisted of 5 μl of FastStart SYBR Green Master Mix (Roche Applied Science), 0.2 μl of each primer (10 μmol/μl), and 1 ng/μl DNA template in a 10 μl volume. The primers BchF and BchR were used for *R. palustris* species-specific quantification, and the 16S rDNA universal primers 340F and 514R were used for total bacterial quantification (Supplementary Table S3). The PCR conditions were as follows: initial denaturation at 95°C for 10 min; 45 cycles of denaturation at 95°C for 15 s, annealing at 60°C for 30 s, and extension at 72°C for 30 s.

### Verification with pot experiments

The pot experiments were conducted using two cherry tomato cultivars (i.e., “Yu Nu” and “Red Pearl”). Tomato seedlings were planted in soil-filled pots (containing approximately 1.0 kg of soil) and grown in a phytotron (Agricultural Experimental Station, National Taiwan University, Taiwan) with natural sunlight at 25/20°C day/night and 80 (±5) % relative humidity. The pot experiments were conducted with the following treatments: (a) organic fertilizer 426 without inoculant (OF+M); (b) organic fertilizer 426 with PS3 inoculant (OF+PS3). Each treatment had five replicates. The organic fertilizer had an N/P/K ratio of 4:2:6 (FWUSOW INDUSTRY CO., LTD., Taiwan). Then, 0.86 g was applied to each pot, and the plants were fertilized in every development stage of tomato (seedlings, flowering, and fruit formation). The dosage of the PS3 inoculant was adjusted to approximately 1.0 × 10^9^ CFU ml^−1^, and 20 ml of bacterial suspension was applied to the topsoil (root surrounding) in each pot. After 95 days of planting, fruits and soils were sampled for analysis as described above.

### Statistical analyses

For plant phenotype and soil environment data, bar plots were generated using GraphPad Prism 6. The statistical analysis was performed based on Welch’s t test. Microbial community analyses were conducted with QIIME2 (Hall and Beiko, 2018). To normalize the sequencing output among samples, we rarefied the OTU table to 16,000 reads per sample (Supplementary Figure S3). Alpha diversity indices, including the observed OTU counts and Shannon index, were calculated by the “qiime diversity alpha” function. The differences in microbial community composition among all treatments were analyzed by principal coordinate analysis (PCoA) based on Bray–Curtis distances. The statistically significant differences between treatments were assessed by permutational ANOVA (PERMANOVA). The log2-fold change coupled with permutation t test (value of *p* < 0.05) was used to identify significantly different biomarkers/OTUs at the genus level among the treatments. The dot plots were illustrated by the R x64 ver 3.5.3 package “DESeq2” (https://www.r-project.org/; Love et al., 2014). In addition, Pearson correlation coefficients were used to analyze the relationships among predominant OTUs, plant growth traits, and soil environments.

## Results

### PS3 inoculation showed beneficial effects on the yields and quality of tomato fruits

**Figure 1:**
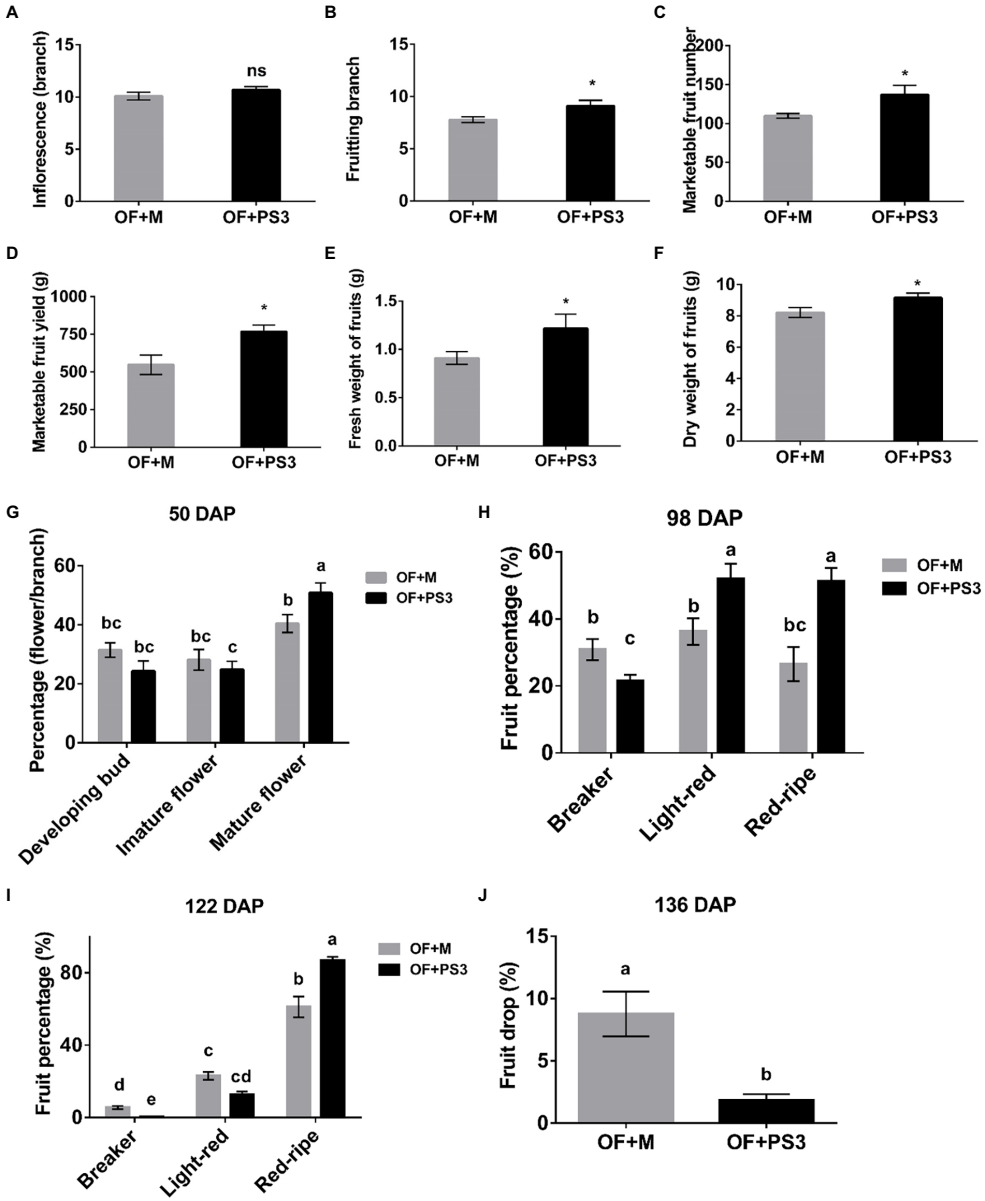
Effects of *Rhodopseudomonas palustris* PS3 on grafted tomato vegetative and reproductive growth traits. **(A)** Number of inflorescence branches. **(B)** Number of fruiting branches. **(C)** Marketable fruit numbers. **(D)** Marketable fruit yield. **(E)** Fresh weight of fruits. **(F)** Dry weight of fruits. **(G)** Flower development for a the first branch at various stages 50 days after planting (DAP). **(H)** Fruit color transformation for the first–sixth branches at 98 DAP. **(I)** Fruit color transformation for the first–sixth branches at 122 DAP. **(J)** Fruit drop percentage for all harvested branches of grafted tomato at 136 DAP. OF + M: applying the plant-based organic fertilizer and new PNSB medium in the soil; OF + PS3: applying plant-based organic fertilizer and PS3 inoculant in the soil. DAP represents days of planting in panels **(G–J)**. All results are expressed as the mean ± SE. The statistical test was based on Welch’s *t*-test for panels **(A–F)** (ns, *p* ≥ 0.05; *, *p* < 0.05) and one-way ANOVA followed by Tukey’s *post hoc* test for panels (**G–J**; letters above means indicate significant differences among treatments at a threshold of *p* < 0.05).

By comparing the PS3-inoculated plants (i.e., OF+PS3) to the control group (i.e., OF+M), we found significant differences in all of the traits measured except for the number of inflorescence branches (Figure 1). Notable effects of PS3 inoculation included increasing the number of fruiting branches by 25% (Figure 1B), the yield of marketable tomato fruit by 37% (Figure 1D), and the fresh weight of individual tomato fruit by 33% (Figure 1E). With regard to the flowering percentage, we found that the value for mature flowers of the OF+PS3 treatment was 10% higher than that of the OF+M treatment at 50 days after planting (50 DAP; Figure 1G). For verification, we performed a pot experiment with tomato seedlings (var. Yu Nu) grown in Akadama soil. As shown in Supplementary Figure S4, we observed that the total flower number of the PS3-treated plants was significantly higher than that of the OF+M treatment.

According to the classification by Qin et al. (2012), we determined the percentage of tomato fruit at the corresponding ripening stages in individual plants based on their surface color (Figures 1H,I). At 98 DAP, those classified as being in the light-red and red ripe stages were 1.3–2.0 times more abundant in the OF+PS3 treatment than in the OF + M treatment (Figure 1H). In contrast, those classified as being in the breaker stage were fewer in the OF + PS3 group. At 122 DAP, most of the fruit had ripened in both groups, and those classified as being in the red ripe stage were 20% more abundant in the OF + PS3 group (Figure 1I). At the end of the harvesting (136 DAP), we calculated the total number of dropped fruit from the first to sixth branches in an individual plant. As shown in Figure 1J, the fruit drop rate in the OF + PS3 group (1.85%) was lower than that in the OF + M treatment (9.6%).

**Figure 2:**
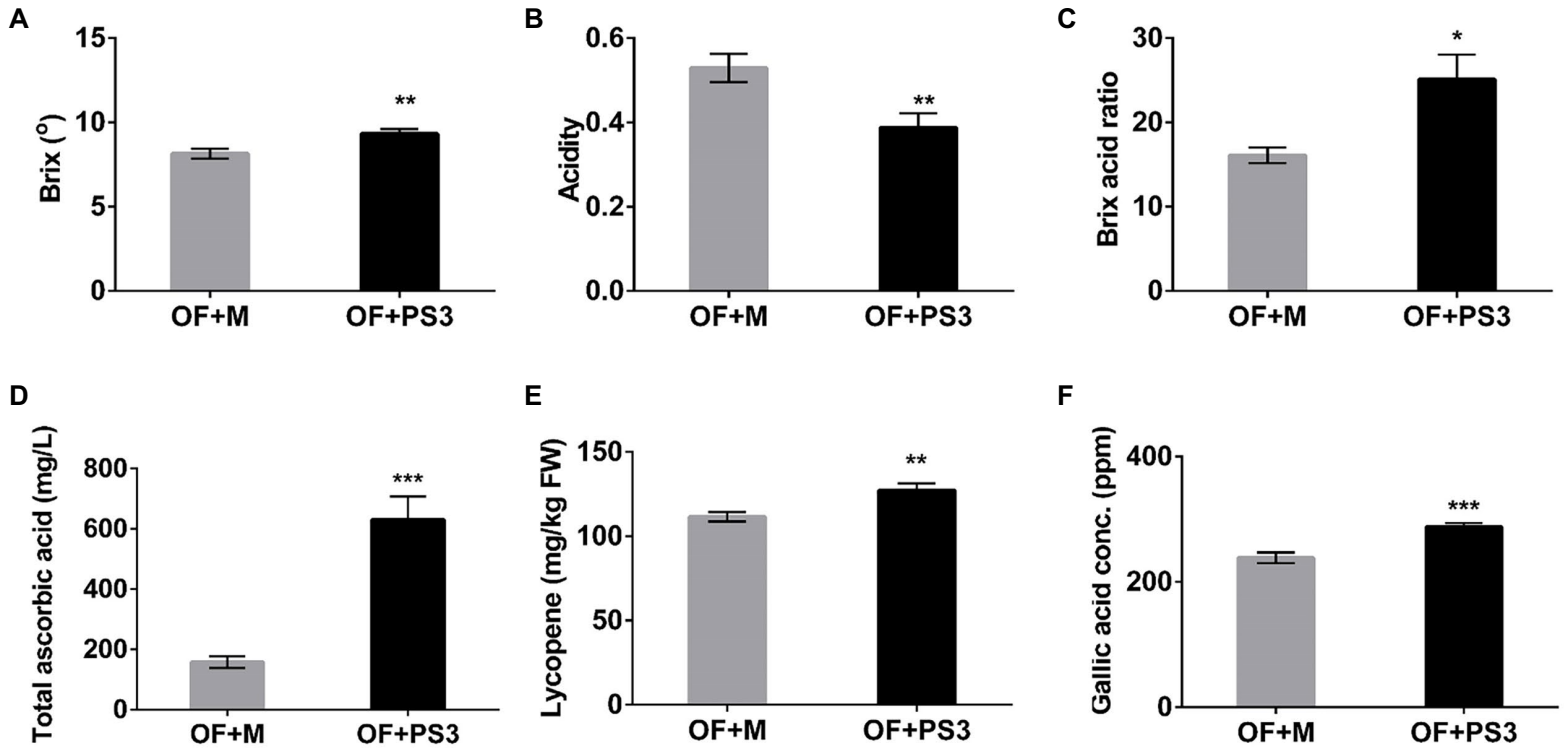
Effects of *Rhodopseudomonas palustris* strain PS3 on grafted tomato harvesting quality at the ripening stage. **(A)** Total soluble solids (°Brix). **(B)** Acidity. **(C)** Brix/acid ratio. **(D)** Total ascorbic acid (mg/L). **(E)** Lycopene contents (mg/kg FW). **(F)** Gallic acid concentration (equivalent to total phenolic content). All results are expressed as the mean ± SE. The statistical test was based on Welch’s *t*-test (*, *p* < 0.05; **, *p* < 0.01; and ***, *p* < 0.005).

The postharvest quality of the tomato fruit was markedly higher in the PS3-inoculated group. The Brix (TSS) and acidity values of the tomato fruit were determined and are shown in Figure 2. The TSS content mainly includes sugars (fructose), and the ratio of sugars to acid (Brix/acid ratio) can act as a simple index of fruit quality (taste; Aoun et al., 2013). As shown in Figure 2, the sweetness and taste of tomato fruit from the OF + PS3 group were significantly increased, by 12 and 38%, respectively (Figures 2A–C). Furthermore, the levels of ascorbic acid (vitamin C), total phenolic compounds, and lycopene were assessed to quantify the antioxidant and nutritional quality of tomato fruit. As shown in Figure 2D, the total ascorbic acid content of tomato treated with PS3 was approximately three times that of the control-treated tomato. The lycopene and total phenolic levels increased by 19 and 16%, respectively (Figures 2E,F).

### PS3 inoculation promoted root growth

**Figure 3:**
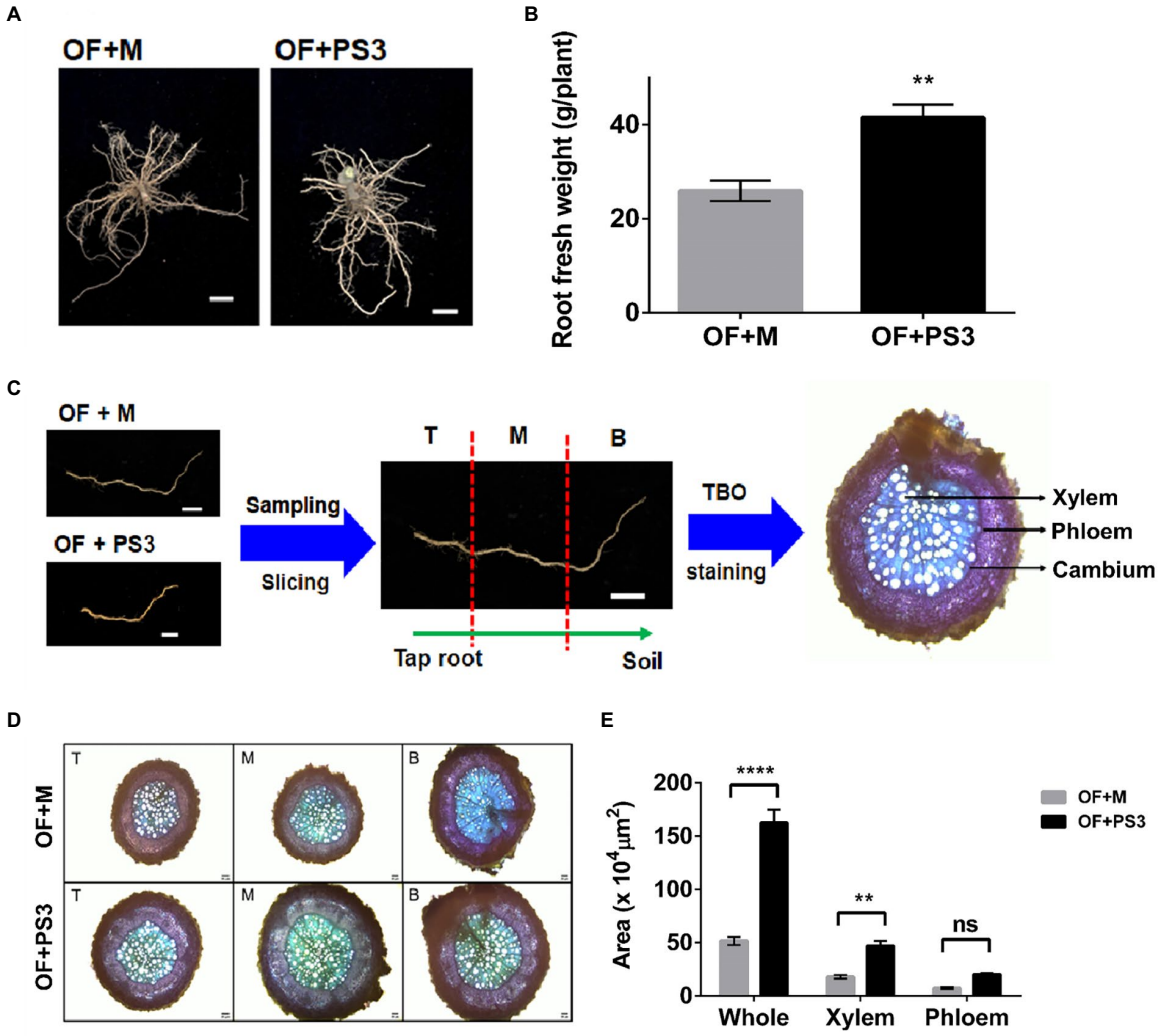
Comparison of the morphological root growth patterns between the control and *Rhodopseudomonas palustris* PS3 inoculation groups. **(A)** Root phenotype. **(B)** Root fresh weight. **(C)** Sampling procedures for light microscopic observation. Sampling and sectioning methods are based on distance from taproot, where T is close to tap root, M distance is midway between taproot and soil, and B is far away from taproot. **(D)** Cross-sections of grafted tomato roots (T, M, and B) stained with Toluidine blue (TBO). **(E)** Average total root area, xylem area, and phloem area. The statistical test was based on Welch’s *t*-test.(ns, *p* ≥ 0.05; ** *p* < 0.01, **** *p* < 0.001). Data are expressed as the mean ± SE.

As shown in Figures 3A,B, the PS3-treated plants showed marked increases in root diameter and biomass (i.e., fresh weight). For microscopic observation, we sliced the lateral roots into three sections (T: root close to taproot, M: root midway between taproot and soil, and B: root far away from taproot; Figure 3C). As shown in Figures 3D,E, the corresponding areas for different sections of the OF + PS3-treated roots were significantly greater than those for sections of the OF + M group, as shown by staining analysis. We noticed that the xylem area increased markedly in the presence of PS3 inoculation (Figures 3D,E), whereas the phloem area of OF + PS3 was slightly larger than that of OF + M, but the difference was not statistically significant (Figures 3D,E).

### PS3 inoculation enhanced soil nutrient availability and soil enzymatic activities

**Figure 4:**
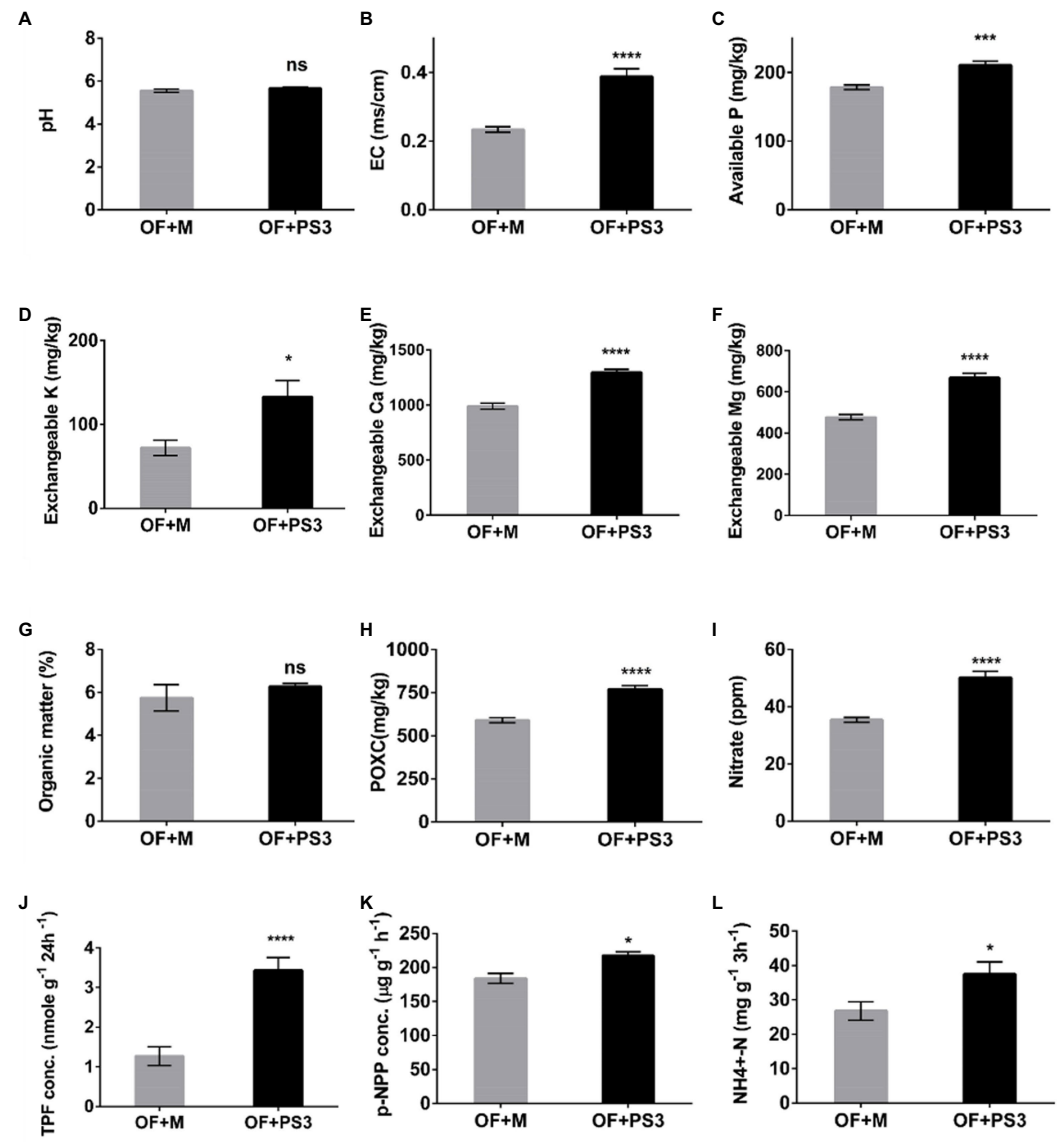
Physicochemical properties and enzyme activities of soil samples under different treatments. **(A)** pH. **(B)** Electroconductivity (EC). **(C)** Available phosphorus (P). **(D)** Exchangeable potassium (Ex.K). **(E)** Exchangeable calcium (Ex.Ca). **(F)** Exchangeable magnesium (Ex.Mg). **(G)** Organic matter (%). **(H)** Available carbon (POXC). **(I)** Nitrate. **(J)** TPF concentration (represented as soil dehydrogenase activity). **(K)** *p*-NPP concentration (represented as phosphatase activity). **(L)** NH4^+^-N (represented as urease activity). TPF, triphenylformazan; *p*-NPP, para-nitrophenylphosphate. The statistical test was based on Welch’s t-test (ns, *p* ≥ 0.05; * *p* < 0.05, *** *p* < 0.005, and **** *p* < 0.001). Data are five biological replicates and expressed as the mean ± SE.

Soil physicochemical properties were affected by PS3 inoculation. As shown in Figure 4, the soil EC and the levels of most available nutrients, such as available P, Ex.K, Ex.Ca, Ex.Mg, and nitrate, were higher in the PS3-inoculated samples, although no significant difference was observed for the total organic matter. However, the active carbon content (POXC) in the OF + PS3 soil (780 mg/kg) was significantly higher than that in the OF+M treatment (600 mg/kg; Figure 4H). Notably, inoculation with PS3 significantly increased the amount of endogenous nitrate in plants (Figure 4I). Overall, inoculation with PS3 improved the available nutrients in the soil and endogenous N content in plants. Furthermore, as shown in Figures 4J–K, the dehydrogenase, phosphatase, and urease activities in the OF+PS3 soil increased by 62, 13, and 36%, respectively.

### Indigenous soil microbial community structure was affected by PS3 inoculation

**Figure 5:**
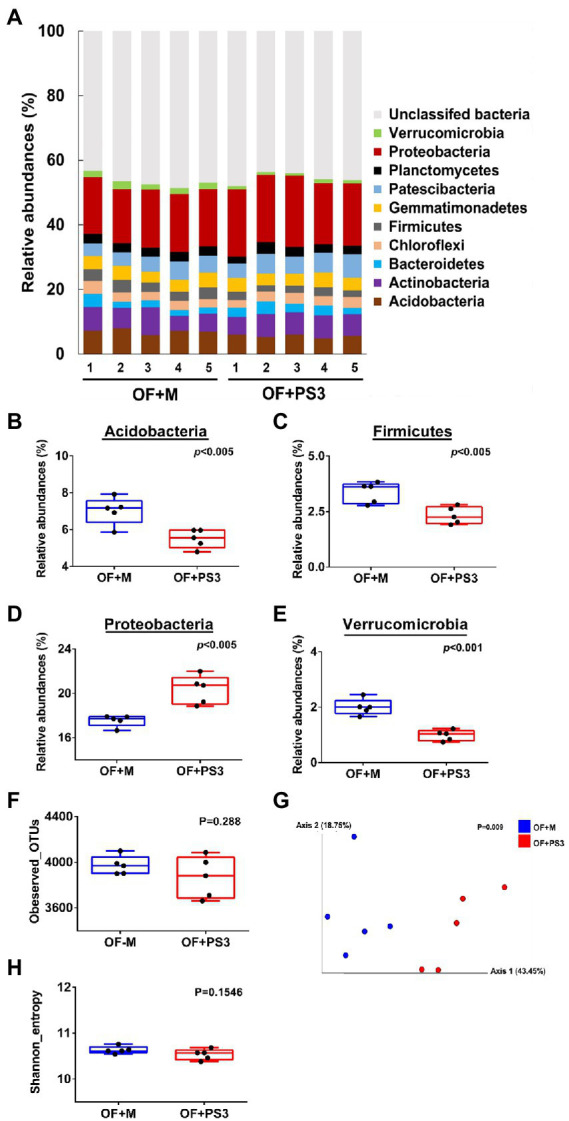
Microbiota composition. Five biological replicates (labeled as 1–5) were used for each set. **(A)** Relative abundance at the phylum level; only those accounting for at least 1.0% are labeled, and the remainder is grouped under “unclassified bacteria.” **(B–E)** Bacterial phyla exhibiting significant differences in relative abundance between different treatments (*p* < 0.05); alpha-diversity includes **(F)** observed OTUs and **(G)** mean species richness (Shannon index); beta-diversity indicated by **(H)** principal coordinate analysis (PCoA) based on weighted UniFrac distance; Blue dots represent full rate organic fertilizer with nutrient medium (OF + M). Red dots indicate full rate organic fertilizer with PS3 inoculum (OF + PS3). A data point denotes one sample or biological repeat. Each treatment consisted of five biological repetitions.

We determined the rhizosphere microbiota composition in the organic tomato farmland by a culture-independent 16S rDNA amplicon survey. A total of 411,722 high-quality sequences were extracted from the samples, and the read counts per sample ranged from 20,274 to 52,813, with an average of 41,177 (Supplementary Table S2). Based on these reads, 51,822 16S rDNA gene operational taxonomic units (OTUs) were identified (Supplementary Table S2). The taxonomic assignment of OTUs indicated that those samples were dominated by 12 bacterial phyla (relative abundances >1.0%; Figure 5A). Among these phyla, Acidobacteria, Firmicutes, Proteobacteria, and Verrucomicrobia showed significant differences in relative abundance between the OF + M and OF + PS3 samples (Figures 5B–E). Proteobacteria were the most abundant phylum, for which OF + PS3 showed a higher relative abundance than OF + M by 22% (Figure 5D). For the other three phyla, the relative abundances were lower in the OF + PS3 groups (Figures 5B,C,E).

The bacterial diversities of the two groups were assessed using observed OTU counts and the Shannon index (Figures 5F–H). The alpha diversity and total bacterial quantification (approximately 6.79 Log CFU g^−1^) between the two groups did not differ significantly (Figures 5F–G; Supplementary Figures S5A,B; Supplementary Table S3). The overall compositional differences were analyzed based on PCoA (Figure 5H), which demonstrated clear distinction between the OF + M and OF + PS3 samples. Notably, the first and second principal axes explained 43.5 and 18.8% of the total variation, respectively. This finding indicated that although the microbiota composition appeared to be similar at the phylum level (Figure 5A), inoculation with PS3 resulted in considerable changes in the soil microbiota composition at the OTU level.

**Figure 6:**
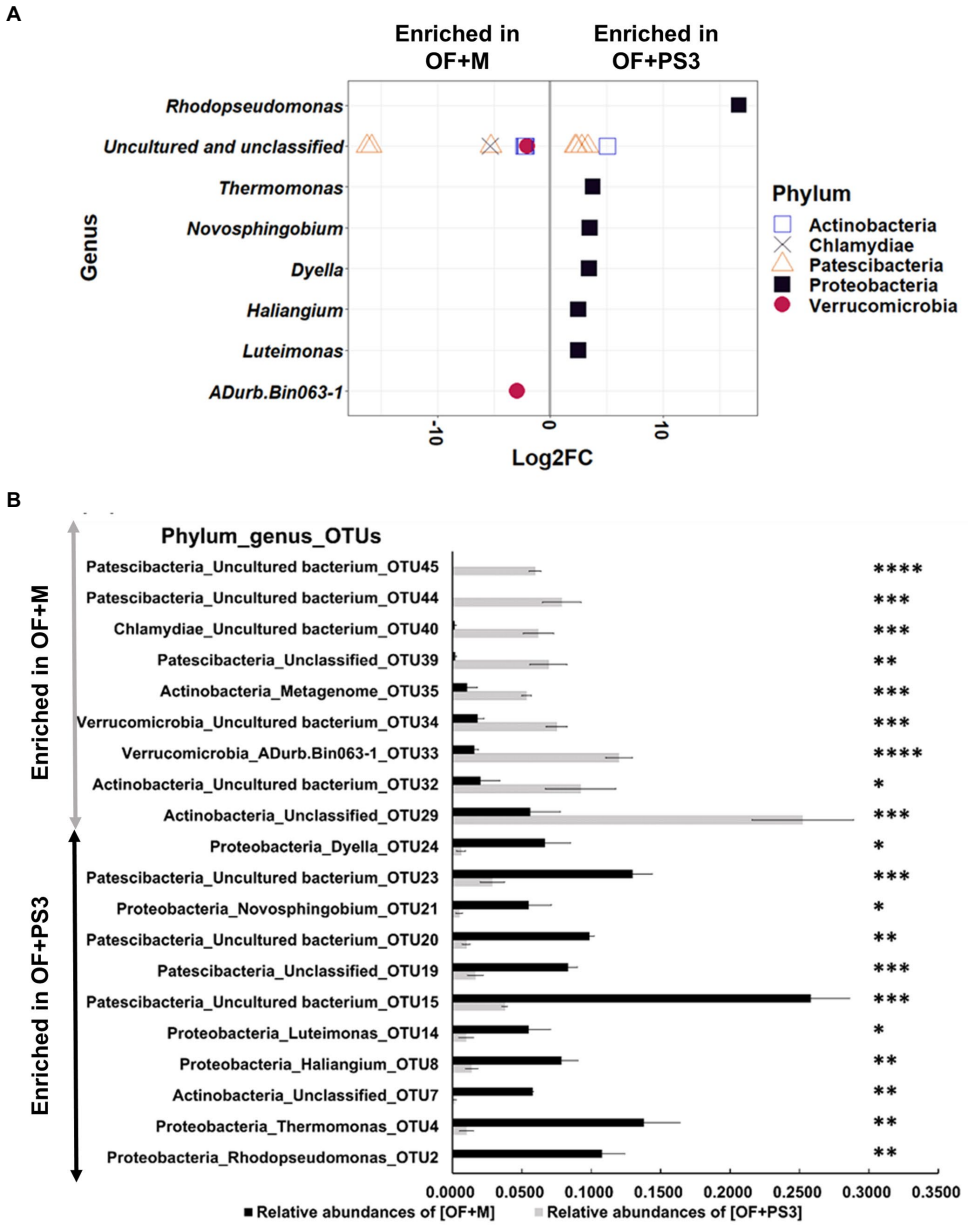
Pairwise comparison. **(A)** Differential abundant of selected OTUs Log_2_-fold change of differentially abundant selected OTUs between the OF + M and OF + PS3 treatments at the phylum and genus levels (*p* < 0.05 and relative abundances >0.05%). OTUs were assigned to genus (*y*-axis) and phylum levels (colors and shapes). Log_2_FC represented as Log_2_-fold change. Negative “Log_2_FC” values (*x*-axis) indicate higher abundance in OF + M samples, and positive values indicate higher abundances in OF + PS3 rhizosphere OF + M: **(B)** Relative abundances of selected individual OTUs in OF + M and OF + PS3 treatments (*p* < 0.05). OF +M: bar in gray, noninoculated control; OF + PS3: bar in black, the inoculation treatments with strain PS3. Significant difference in the relative abundances (≥0.05) of samples based on permutation *T*-test (ns, *p* > 0.05; * *p* < 0.05, ** *p* < 0.01, *** *p* < 0.005, **** *p* < 0.001). Each treatment consisted of five biological repetitions.

Based on the log2-fold change and permutation *t* test of the relative abundance of the OTUs, 46 OTUs were selected as representative OTUs from the OF + M to OF + PS3 groups (Supplementary Table S5). After eliminating 26 OTUs classified only at the domain level, 20 OTUs representing eight genera and five phyla were identified (Figures 6A,B). Among these selected OTUs, 11 OTUs were identified as significant OTUs in the OF + PS3 group, including those belonging to Proteobacteria (black square filled), Patescibacteria (triangle open), and Actinobacteria (square open; Figure 6A). We noticed that the genus *Rhodopseudomonas* had the highest log2-fold change (i.e., 16.71) in the OF + PS3 group (Figure 6A). This finding is consistent with the expectation and indicates long-term survival of the PS3 inoculant in the soil. Furthermore, we applied qPCR with a species-specific primer set to calculate the abundance of *R. palustris*. Approximately 10^7^ CFU/g soil of *R. palustris* was detected in the soil sample of the OF + PS3 treatment, while it was not detected in the OF + M samples (Supplementary Figures S5C,D; Supplementary Table S3). On the other hand, nine selected OTUs were more abundant in the OF+M samples, including those belonging to Patescibacteria (triangle, open), Verrucomicrobia (red circle, filled), Actinobacteria (square, open), and Chlamydiae (cross; Figure 6A).

At the OTU level, we found that OTU4 (genus *Thermomonas*), OTU8 (*Haliangium*), OTU21 (*Novosphingobium*), OTU14 (*Luteimonas*), and OTU24 (*Dyella*) were more abundant in the OF + PS3 samples (Figure 6B). In contrast, OTU44 (unclassified), OTU45 (unclassified), OTU40 (unclassified), OTU39 (unclassified), OTU35 (unclassified), OTU34 (unclassified), OTU33 (ADurb.Bin063-1), and OTU32 (unclassified) were more abundant in the OF + M samples (Figure 6B).

### Correlation between tomato harvesting parameters and soil microbial community composition

**Figure 7:**
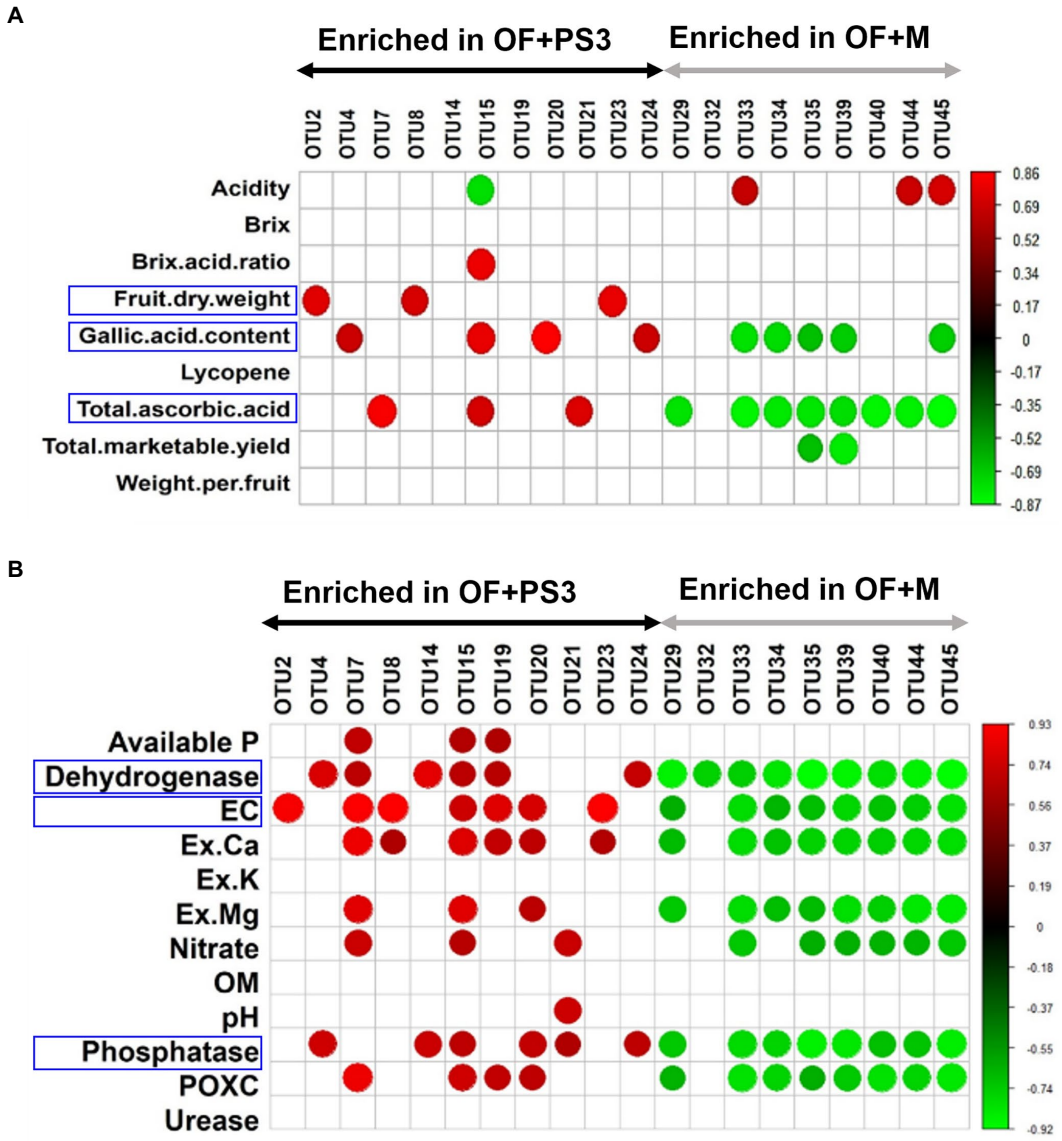
Pearson correlation between the relative abundance of individual microbial groups and plant/soil characteristics. **(A)** Selected OTUs versus tomato harvesting characteristics. **(B)** Selected OTUs versus soil properties. The red and green nodes represent the positive and negative Pearson’s correlation coefficients (*r*), considering a *p* value <0.05, respectively. Each treatment consisted of five biological repetitions.

An analysis of Pearson correlation was conducted to evaluate the relationship between the postharvest parameters of tomato and the relative abundance of individual phyla (relative abundances >1.0%; Supplementary Figure S7). Patescibacteria and Proteobacteria exhibited positive correlations with postharvest or soil parameters (Supplementary Figure S7); also, the majority of selected OTUs were found to belong to these two phyla (Figures 7A,B). Therefore, we examined the relationship between various parameters and the relative abundance of these selected bacterial OTUs for further investigation (Figures 7A,B). Most of the selected OTUs enriched in the OF + PS3 samples were significantly associated with the gallic acid (total phenolic) content, fruit dry weight, and total ascorbic acid content (Figure 7A). Among these, OTU2, OTU8, and OTU23 were positively correlated with fruit dry weight, and OTU4, OTU15, OTU 20, and OTU24 showed positive correlations with the gallic acid content. OTU7, OTU15, and OTU21 were positively correlated with the total ascorbic acid content (Figure 7A). We noticed that OTU15 was positively associated with most postharvest quality parameters (i.e., Brix/acid ratio, flavonoids, and total ascorbic acid) and negatively correlated with acidity (Figure 7A). On the other hand, most of the selected OTUs enriched in the OF+M samples were negatively associated with the gallic acid content and total ascorbic acid content (Figure 7A). Among these, OTU32, OTU33, OTU35, OTU39, and OTU45 were negatively correlated with the gallic acid content. OTU35 and OTU39 were negatively associated with the total marketable yield. OTU33, OTU44, and OTU45 were positively correlated with acidity. These results indicated that some of the selected OTUs exhibited apparent effects on plant harvesting parameters, especially fruit quality.

Moreover, as shown in Figure 7B, 80% of the OTUs were correlated with soil environmental factors, such as EC, dehydrogenase activity, and phosphatase activity. OTU2 exhibited a high positive correlation with EC. OTU4, OTU14, and OTU24 were positively correlated with dehydrogenase and phosphatase activities. OTU7 and OTU15 exhibited high abundances and positive correlations with most soil parameters. OTU8 and OTU23 were positively correlated with EC and Ex.*Ca.* OTU20 was positively associated with EC, Ex.Ca, Ex.Mg, phosphatase, and POXC. Most of the selected OTU abundances in the OF+M samples were negatively correlated with soil parameters, especially dehydrogenase, EC, Ex. Ca, Ex. Mg, nitrate, phosphate, and POXC (*p* < 0.05). Among the environmental factors, dehydrogenase showed a strong negative correlation with the selected OTUs in the OF + M samples (*r* = −0.89, *p* = 6 × 10^−4^).

### The pot experiment reproduced the correlation between the selected parameters and selected genera

**Figure 8:**
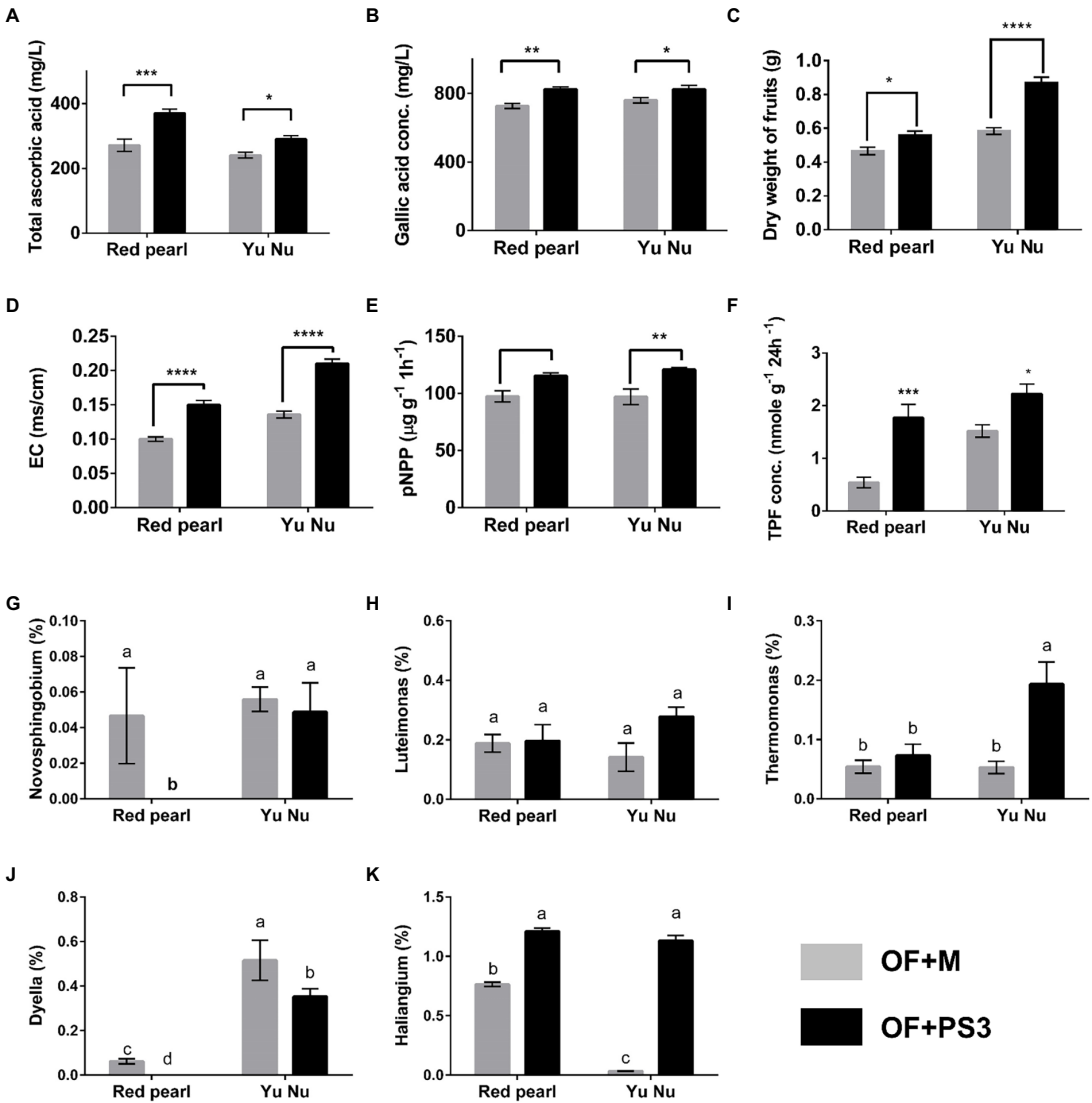
Verifying the selected parameters and potential genera by pot experiments with two tomato cultivars (Red pearl and Yu Nu) and soil collected from the aforementioned organic field. **(A)** total ascorbic acid (vitamin C content); **(B)** gallic acid concentration (total phenolic content); **(C)** dry weight of fruits; **(D)** soil electroconductivity (EC); **(E)** p-NPP concentration (soil phosphatase activity); **(F)** TPF concentration (soil dehydrogenase activity); Relative abundances of **(G)** *Novosphingobium* (%); **(H)** *Luteimonas* (%); **(I)** *Thermomonas* (%); **(J)** *Dyella* (%); and **(K)** *Haliangium* (%). Data are expressed as the mean ± SE. The statistical test was based on Welch’s t-test for panels **(A–F)** (ns, *p* > 0.05,* *p* < 0.05, ** *p* < 0.01, *** *p* < 0.005, and **** *p* < 0.001) and one-way ANOVA followed by Tukey’s *post hoc* test for panels **(G–K)** (letters above means indicate significant differences among treatments at a threshold of *p* < 0.05). Each treatment consisted of five biological repetitions.

To verify the correlation between the harvest index, soil properties, and the associated selected genus, we performed a pot experiment with better-controlled growth conditions compared to the field trial. The pot experiment used two tomato cultivars (Red Pearl and Yu Nu) and soil collected from the aforementioned organic field. As shown in Figures 8A–C; Supplementary Figure S6, the fruit dry weight, total phenolic content, root weight, and ascorbic value of the PS3-treated tomato fruit were increased by approximately 20, 33, 50, and 6%, respectively, in comparison with those in the OF + M group. Additionally, the EC, soil dehydrogenase activity, and phosphatase activity were all higher in the OF + PS3 soil (Figures 7, 8D–F). It is noteworthy that the increasing trends were similar for both cultivars, indicating the robustness of our findings. We performed 16S rDNA microbiota analysis after harvest and highlighted five dominant bacterial genera in the PS3-treated soil as mentioned above (Figure 6). We found that the relative abundance of the bacterial genus *Haliangium* increased significantly in the presence of PS3 in both cultivars (Figure 8K). The *Thermomonas* abundance was significantly higher in the presence of PS3 in the Yu-Nu cultivar (Figure 8I). On the other hand, the other three bacterial genera (*Novosphingobium*, *Dyella,* and *Luteimonas*) showed no significant difference in abundance in either cultivar following PS3 inoculation (Figures 8G,H,J).

## Discussion

**Figure 9:**
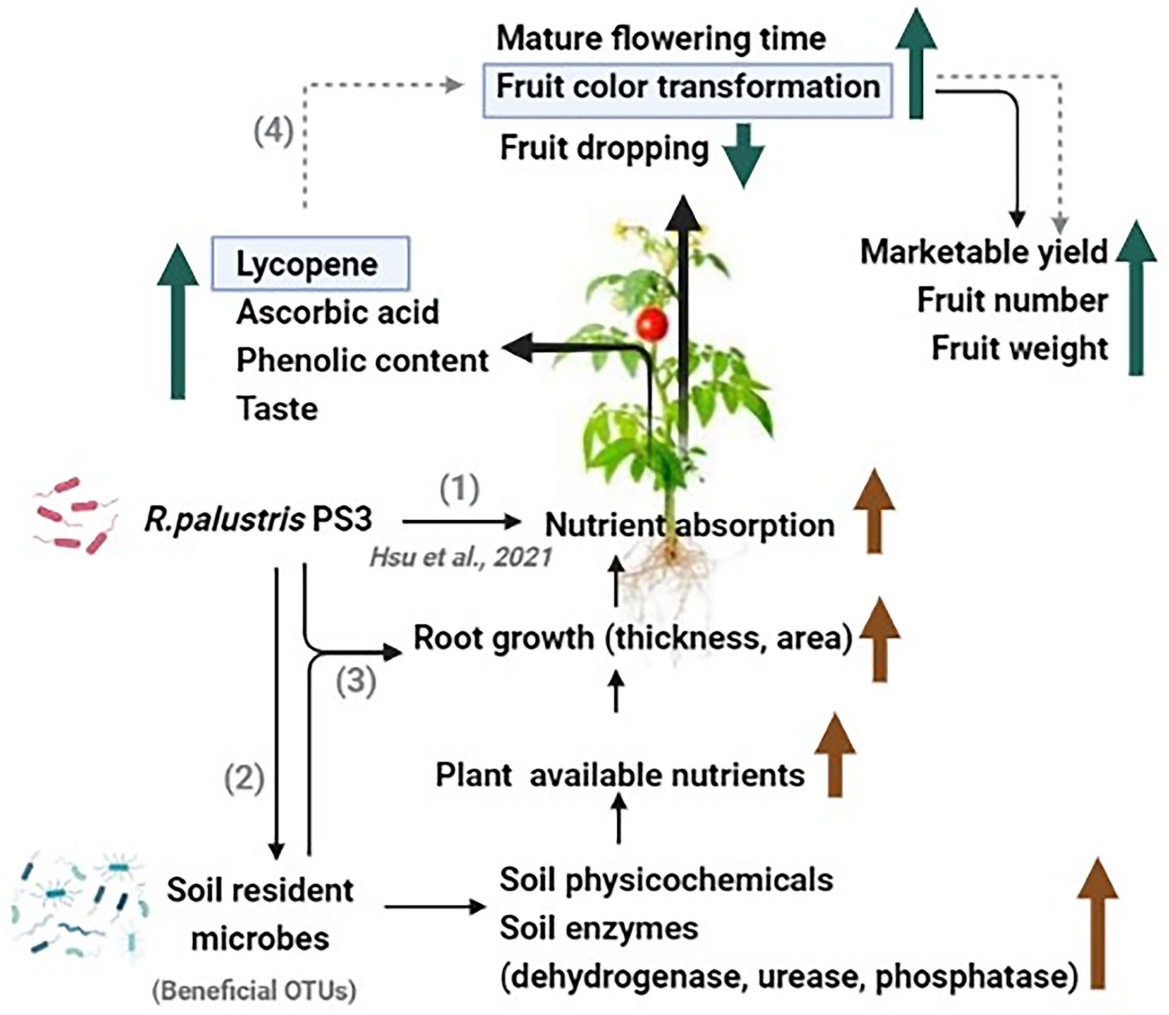
A schematic diagram proposed to explain the potential mechanism by which *Rhodopseudomonas palustris* PS3 could affect tomato development and quality under organic farming systems. (1) *Rhodopseudomonas palustris* PS3 improves the nutrient uptake of plants (Hsu et al., 2021). (2) *Rhodopseudomonas palustris* PS3 promotes fruit growth and quality by altering the soil microbiome, and enhancing soil health and soil enzyme activities. (3) *Rhodopseudomonas palustris* PS3 was applied alone or in combination with other beneficial microbes to enhance root growth and nutrient cycling. (4) *Rhodopseudomonas palustris* PS3 improves fruit color transformation by increasing the lycopene content, thereby enhancing the marketable yield (in prediction).

The PS3 inoculation exhibited notable effects in improving several key agricultural traits of tomato fruits in this study (Figures 2, 3), including marketable fruit yield, vitamin C content, phenolic content, and lycopene concentration, etc. According to our previous studies, the presumed mechanisms include (1) biofertilization for increasing plant-available nutrients in soil and (2) biostimulation for stimulating plant nutrition use efficiency (reviewed by Lee et al., 2021a). In this study, PS3 inoculation enhanced the soil physicochemical traits and enzymatic activities in the organic field (Figure 4). Since recent studies have revealed that PGPRs could affect soil physicochemical properties by interacting with microbial communities (Bhattacharyya et al., 2018; Mickan et al., 2021), we also investigated the soil microbiota composition in this study based on a high-throughput culture-independent approach. We found that several bacterial genera (*Novosphingobium*, *Luteimonas*, and *Thermomonas*) related to soil nutrient cycling and soil health had relatively higher abundances in the PS3-inoculated soil (Figures 5, 6). Diazotrophic *Novosphingobium* spp. exhibit distinctive endophytic colonization and have various PGP characteristics, such as phosphorus solubilization and nitrogen fixation (Smit et al., 2012; Rangjaroen et al., 2017). *Luteimonas* spp. have been reported to possess the ability to degrade complex organic compounds, resulting in an increase in nutrient uptake for nitrogen or phosphorus in plants to promote early plant development (O’brien, 2016; Kohout, 2019; Lin et al., 2020). *Thermomonas* spp. are denitrification and nitrogen fertilizer-associated bacteria that are responsible for saccharase activity (O’brien, 2016). We hypothesized that the beneficial effects on tomato exerted by PS3 inoculation could be attributed to the direct stimulatory effect of PS3 on plant physiology and the indirect stimulatory effect *via* interactions with microbial communities to elevate the available soil nutrients (Figure 9).

We noticed that the fruit drop percentage of preharvest OF+PS3-treated tomato (9%) was significantly lower than that of OF+M-treated tomato (2%; Figure 1J). Premature fruit drop is often associated with abiotic stresses, such as extreme temperature, water stress, or low light intensity (Lee et al., 2008, 2021b). Based on the data from historical weather observations and statistics provided by the Central Weather Bureau of Taiwan, the tomato plants experienced dramatically insufficient sunlight during their fruit set period (December 2020) compared with the past growing seasons (Supplementary Table S4). This may explain the high percentages of premature fruit drop, particularly in the control group. Premature fruit drop or fruit abscission is generally regulated by phytohormones, such as ethylene and abscisic acid (ABA) and signal transduction pathways (Botton et al., 2011). However, very few studies have discussed the molecular mechanisms by which PGPRs prevent fruit drop. Lee and colleagues reported that the expression levels of cell wall degradation and abscisic acid signaling genes were all upregulated during abscission (Lee et al., 2021b). Belimov and colleagues reported that treating rice (*Oryza sativa*) seedlings with an abscisic acid-metabolizing rhizobacterium, *Novosphingobium* sp. P6W, could decrease or alter the ABA concentrations in plants (Belimov et al., 2014). We noticed that the genus *Novosphingobium* dominated in the PS3-treated soil (Figure 6). Accordingly, we speculated that the beneficial effect of PS3 was not limited to the direct beneficial effects of PGP (i.e., improve N uptake), but also to the regulation of phytohormone responses *via* the soil microbiota.

Several studies have indicated that PGPR inoculation may regulate the composition of host rhizobacterial communities (Zhang et al., 2019a,b), which are involved in plant growth promotion (Gadhave et al., 2018; Wang et al., 2018a). This study found that inoculation with PS3 in organic tomato farmland led to a higher relative abundance of Proteobacteria (Figure 5D). Proteobacteria are related to a wide range of biological activities associated with the cycling of carbon, nitrogen, sulfur, and other elements (Spain et al., 2009). Smit et al. (2001) proposed that the ratio between the abundances of Proteobacteria and Acidobacteria (P/A) could indicate the general nutrient status of soils. Low P/A ratios (0.16–0.46) suggest that the soils are oligotrophic, while high ratios (0.87 or above) indicate that they are copiotrophic (Smit et al., 2012; Shen and Lin, 2021). As shown in Figures 5B,D, the average P/A ratios in the OF+M control and the OF+PS3 treatment were 2.46 and 3.64, respectively, suggesting an improvement in soil nutritional status after PS3 inoculation. Furthermore, since Proteobacteria were the most dominant OTUs in the PS3-treated soil (Figure 6), we deduced that the PS3 inoculant acted synergistically with the nutrient cycle-related phylum Proteobacteria to improve the biomass and quality of the tomato plant.

As shown in Figure 7, the selected OTUs in the PS3-treated soil samples of the organic field revealed positive relationships with fruit dry weight, total phenolic content, total ascorbic acid content, soil dehydrogenase activity, phosphatase activity, and EC. To verify the correlation, we conducted pot experiments with seedlings of two tomato cultivars (i.e., Red Pearl and Yu Nu) and the same soil collected from the organic field (Figure 8). We found that PS3 inoculation significantly improved the harvest qualities of tomato fruit and the soil characteristics, consistent with the observations from the field experiments. Since the soil EC value is positively correlated with the nutrient availability of the soil (Heiniger et al., 2003), PS3 inoculation could improve soil fertility. In addition to nutrients, we also found that the activities of some soil enzymes, such as dehydrogenase and phosphatase, were significantly increased (Figures 4J,K). Since soil enzyme activity is regarded as an indicator of microbial activity (Quilchano and Marañón, 2002; Gu et al., 2009; Salazar et al., 2011), we hypothesize that PS3 inoculation can stimulate microbial activity in the soil.

One notable difference between the field trial and pot experiments was that while five genera were found to have higher relative abundance in the field upon PS3 inoculation (Figure 6B), only *Haliangium* had significantly higher abundance in the pot experiment (Figure 8K). With regard to the other four genera (i.e., *Novosphingobium, Dyella, Luteimonas,* and *Thermomonas*), the differences between the field trial and pot experiment may be explained by multiple factors, particularly temperature. It has been reported that temperature or season is the key factor determining the relative abundance or composition of soil bacteria (Frindte et al., 2019; Wang et al., 2022; Zhelezova et al., 2022). We conducted the pot experiments in a phytotron under well-controlled temperature conditions (25/20°C day/night and 80 (±5) % relative humidity). In contrast, the temperature conditions in the organic field were dynamic, including fluctuations in the diurnal temperature range (min: 14°C; max: 29°C) and moisture. It is likely that *Haliangium* was not easily affected by the dramatic change in climate. In addition, we noticed that even though we inoculated PS3 with different tomato cultivars (Red Pearl and Yu Nu), the relative abundance of *Haliangium* remained high. *Haliangium* spp. have been proposed to inhibit fungal pathogens in plants by producing particular secondary metabolites (e.g., haliangicins; Sun et al., 2016; Wang et al., 2018b). Additionally, *Haliangium* spp. have been regarded as indicators of soil quality and hubs of PGPRs (Zhou et al., 2018; Shang and Liu, 2021; Wolińska et al., 2022). Based on the findings and literature, we propose that *Haliangium* can be a potential indicator for determining the efficiency of PS3 inoculation in soil, and this needs to be further verified in various fields and studied on microbe-microbe interactions.

## Conclusion

This study enhances our understanding of the effects of beneficial phototrophic bacteria on plant growth, soil physicochemical properties, and the composition of microbial communities in the soil of organic tomato farms. According to our results, inoculation with *R. palustris* strain PS3 significantly improved the yield and quality of tomato plants under organic farming practices. Several bacterial groups associated with nutrient cycling exhibited higher relative abundance after PS3 inoculation. These differences were positively correlated with the growth characteristics and harvest quality of tomato, suggesting that a favorable rhizosphere bacterial community may contribute to improvements in fruiting and yield. Therefore, inoculation with PGPRs, such as the strain PS3 characterized in this study, can provide a sustainable way of improving the health of plants and soil in modern agriculture.

## Data availability statement

The data presented in the study are deposited in the online repository, accession number PRJNA865887, http://www.ncbi.nlm.nih.gov/bioproject/865887.

## Author contributions

S-KL, C-HK, and C-TL: conceptualization. S-KL: experiment and data analysis. S-KL and C-TL: writing–original draft preparation. C-HK, M-SC, and Z-YH: review and editing and American Journal Experts English editing service. C-HK and C-TL: supervision. C-TL: funding acquisition. All authors contributed to the article and approved the submitted version.

## Funding

This study was supported by grants from the Ministry of Science and Technology (108-2313-B-002-058-MY3, MOST 109-2321-B-005-027) and Academia Sinica. Computational resources were provided by the Institute of Plant and Microbial Biology, Academia Sinica. This article was subsidized for English editing by National Taiwan University under the Excellence Improvement Program for Doctoral Students (108-2926-I-002-002-MY4), sponsored by Ministry of Science and Technology, Taiwan.

## Conflict of interest

The authors declare that the research was conducted in the absence of any commercial or financial relationships that could be construed as a potential conflict of interest.

## Publisher’s note

All claims expressed in this article are solely those of the authors and do not necessarily represent those of their affiliated organizations, or those of the publisher, the editors and the reviewers. Any product that may be evaluated in this article, or claim that may be made by its manufacturer, is not guaranteed or endorsed by the publisher.
