# Supplementary material for: A photosynthetic bacterial inoculant exerts beneficial effects on the yield and quality of tomato and affects bacterial community structure in an organic field
Source: Front Microbiol. 2022 Aug 29;13:959080. doi: 10.3389/fmicb.2022.959080 (PMC9479686; doi:10.3389/fmicb.2022.959080)

**Supplementary material for**

**A Photosynthetic bacterial inoculant exerts beneficial effects on the yield and quality of tomatoes and affects bacterial community structure in organic field**

Sook-Kuan Lee^1^, Ming-Shu Chiang^2^, Zeng-Yei Hseu^3^, Chih-Horng Kuo^4^*, Chi-Te Liu^1,3,5^*

^1^Institute of Biotechnology, National Taiwan University, No.81, Chang-Xing St., Taipei 106, Taiwan

^2^Department of Agronomy, National Taiwan University, No.1, Sec. 4, Roosevelt Rd., Taipei 106, Taiwan

^3^Department of Agricultural Chemistry, National Taiwan University, No.1, Sec. 4, Roosevelt Rd., Taipei 106, Taiwan

^4^Institute of Plant and Microbial Biology, Academia Sinica, No.128, Sec. 2, Academia Rd., Taipei 115, Taiwan

^5^Agricultural Biotechnology Research Center, Academia Sinica, No.128, Sec. 2, Academia Rd., Taipei 115, Taiwan

^*^Authors to whom correspondence should be addressed.

CHK: chk@gate.sinica.edu.tw; CTL: chiteliu@ntu.edu.tw.

**(a)**


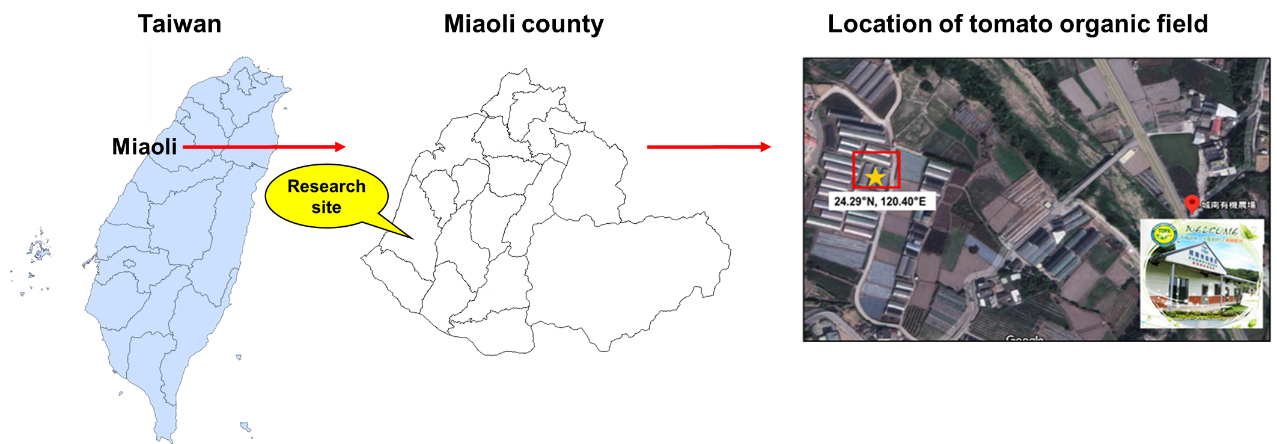


**(b)**
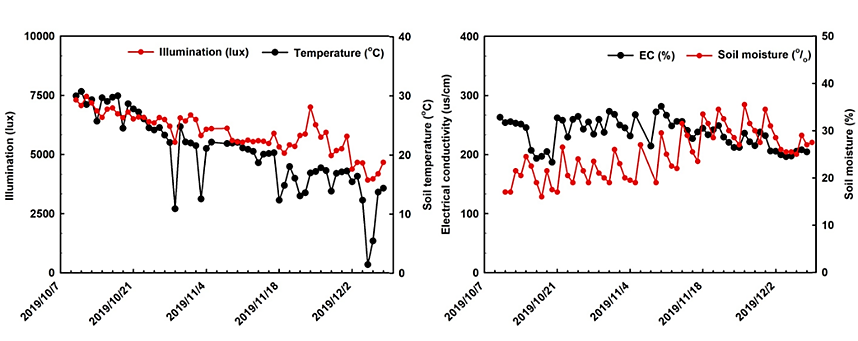


**Fig. S1.** Information on the experimental field in the Town-South organic farm. (a) The farm is located in Tong Hsiao, Miaoli County, Taiwan (24.29°N, 120.40°E). (b) Climate (illumination and temperature) and soil conditions (electroconductivity and moisture) during tomato cultivation.

**(a)**


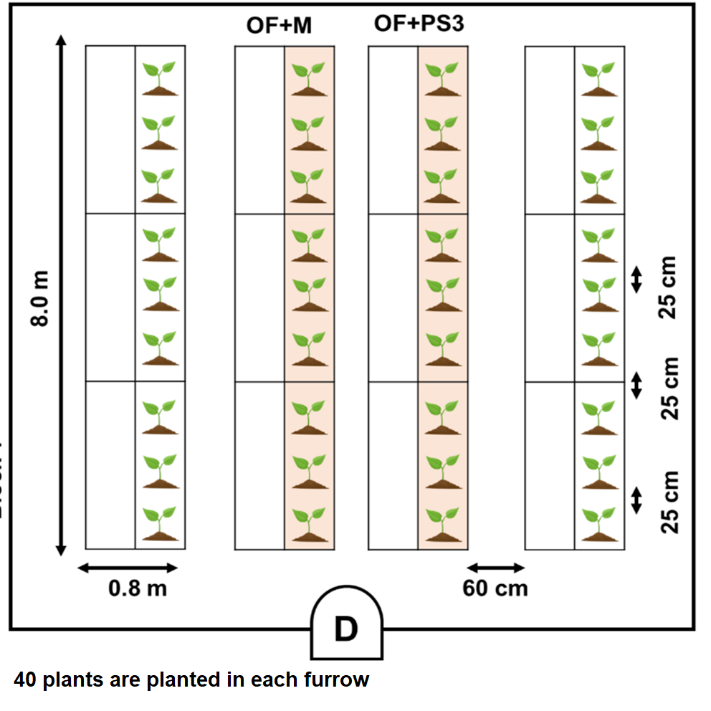


**(b)**


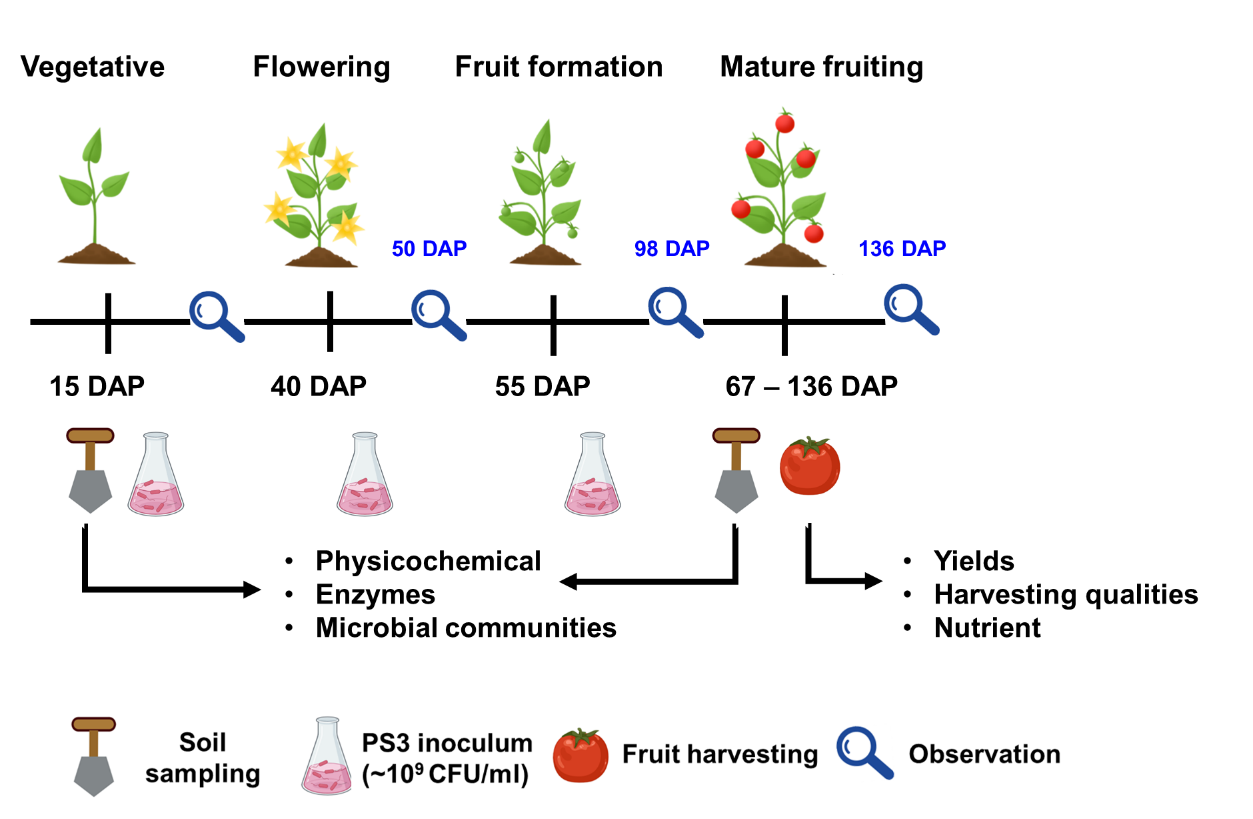


**Fig. S2.** Timeline of inoculation, soil sampling, and harvesting points in the field. Medium or PS3 broth was inoculated into the soil during the following four growth stages: seedling, flowering, fruit formation, and mature fruiting. Soil sampling and tomato harvesting were carried out from 67 days of planting until the final harvest period. DAP is an abbreviation of days after planting.


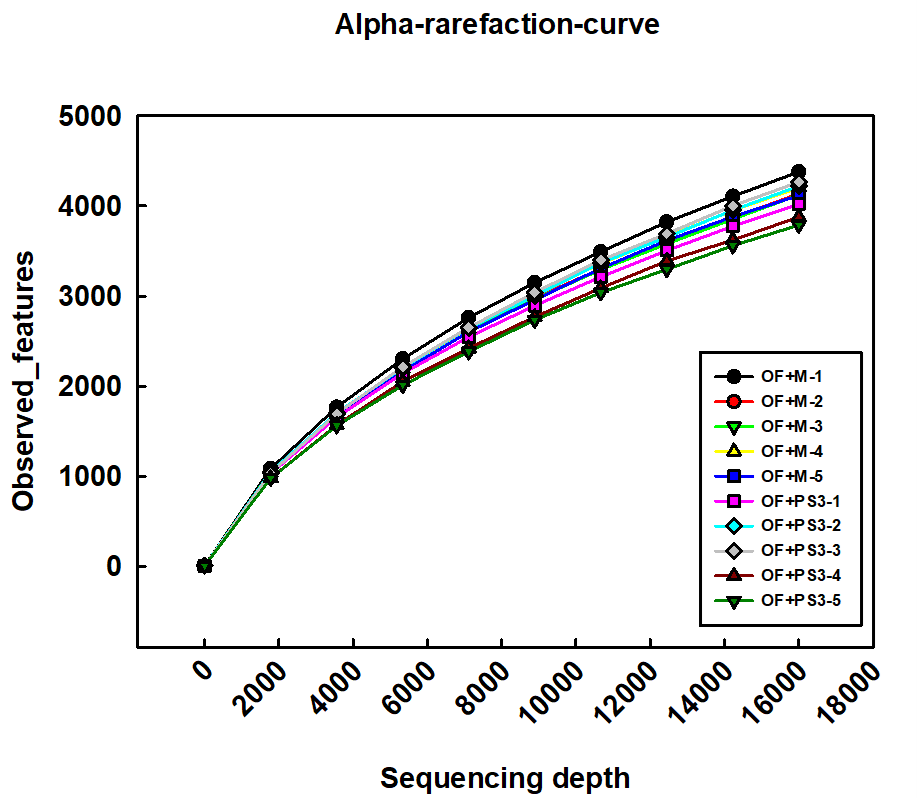


**Fig. S3.** Rarefaction curve of Illumina 16S rDNA amplicon sequencing for the 10 samples.

**
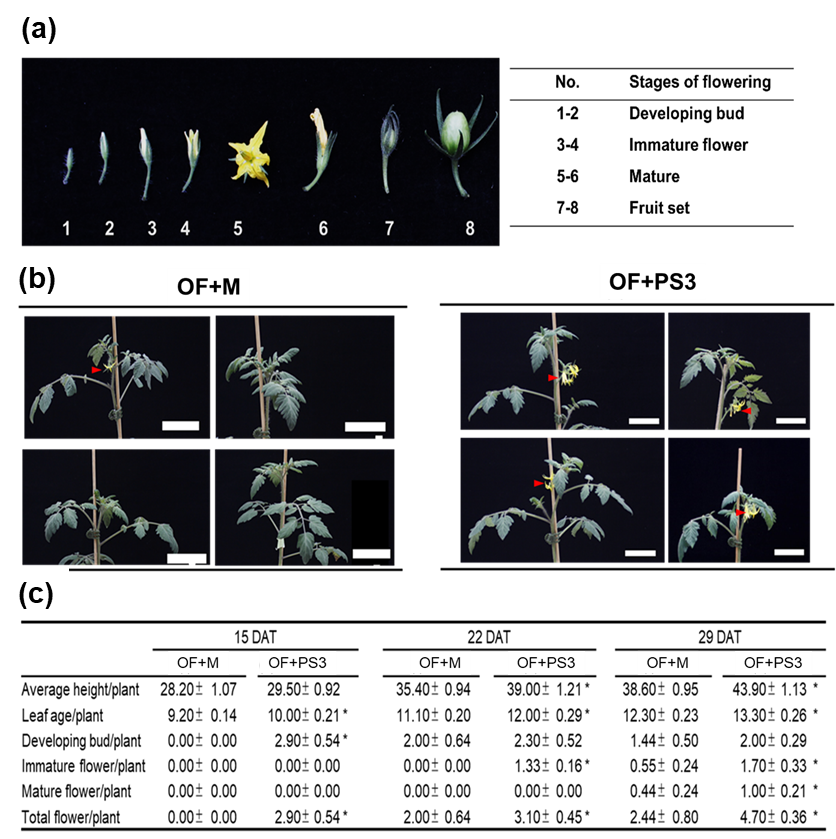
**

**Fig. S4.** Effects of *R. palustris* strain PS3 on vegetative growth and flowering response of tomato in the pot experiment (Akadama soil). (a) Developmental stages of tomato flowers. (b) Phenotypic and morphological comparison between the without inoculation (OF+M) and with inoculation (OF+PS3) treatments (n=4). (c) Response of treatments to various vegetative growth parameters and flower development stages. This experiment was performed in a phytotron (Agricultural Experimental Station, National Taiwan University, Taipei, Taiwan) with natural sunlight at 25/20 °C day/night and 80 (±5) % relative humidity. The cherry tomato var. Yu Nu was used as plant material. Akadama soil was used to test the effect of PS3 on grafted tomato without abiotic disturbance due to its status as nutrient-deficient and sterile soil in its environment. Data are represented as the value ± standard error. Asterisks indicate the statistical significance of individual parameters of OF+PS3 compared with those of OF+M.


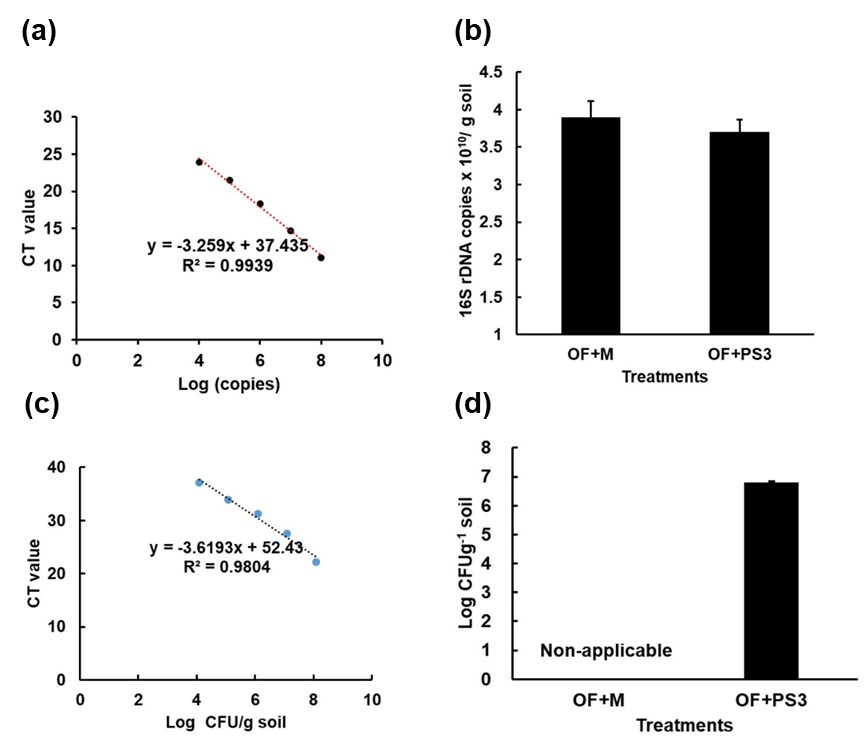


**Fig. S5.** Detection of the total bacterial 16S load and *R. palustris* in different soil samples. (a) Standard curve for total bacterial quantification using universal primers 341F/518R. (b) Comparison of 16S rDNA copy numbers between the OF+M and OF+PS3 treatments. (c) Standard curve for total bacterial quantification using the BchJ gene primers. (d) Comparison of *R. palustris* between the OF+M and OF+PS3 treatments. Data represent the mean of five biological repeats. The cycling program included a 10-min incubation at 95 °C followed by 40 cycles of 95 °C for 15 s, 60 °C for 30 s, and one cycle of 72 °C for 30 s. Melting curve analysis of the PCR products was performed to verify reaction specificity using LightCycler 480 Software v1.5.0 (Roche Applied Science).

**
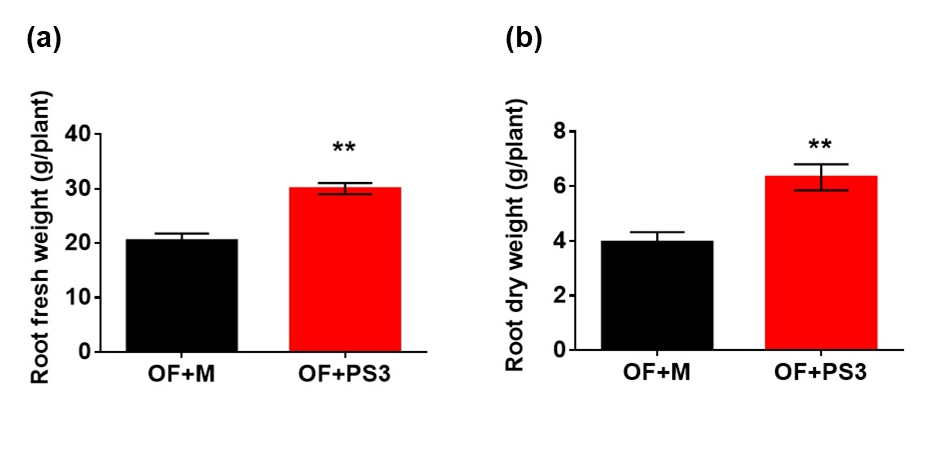
**

**Fig. S6.** Fresh weight (a) and dry weight (b) of tomato (Red pearl) after cultivation in the pot experiment (n = 5). OF+M: applying the plant-based organic fertilizer and new PNSB medium; OF+PS3: applying plant-based organic fertilizer and PS3 inoculant in soil. Asterisks indicate the statistical significance of individual parameters of OF+PS3 compared with those of OF+M, as determined by Welch's t test (ns represented as nonsignificant, * *P* < 0.05, ** *P* <0.01, *** *P* < 0.005, **** *P* < 0.001). Data are expressed as the mean ± SE.


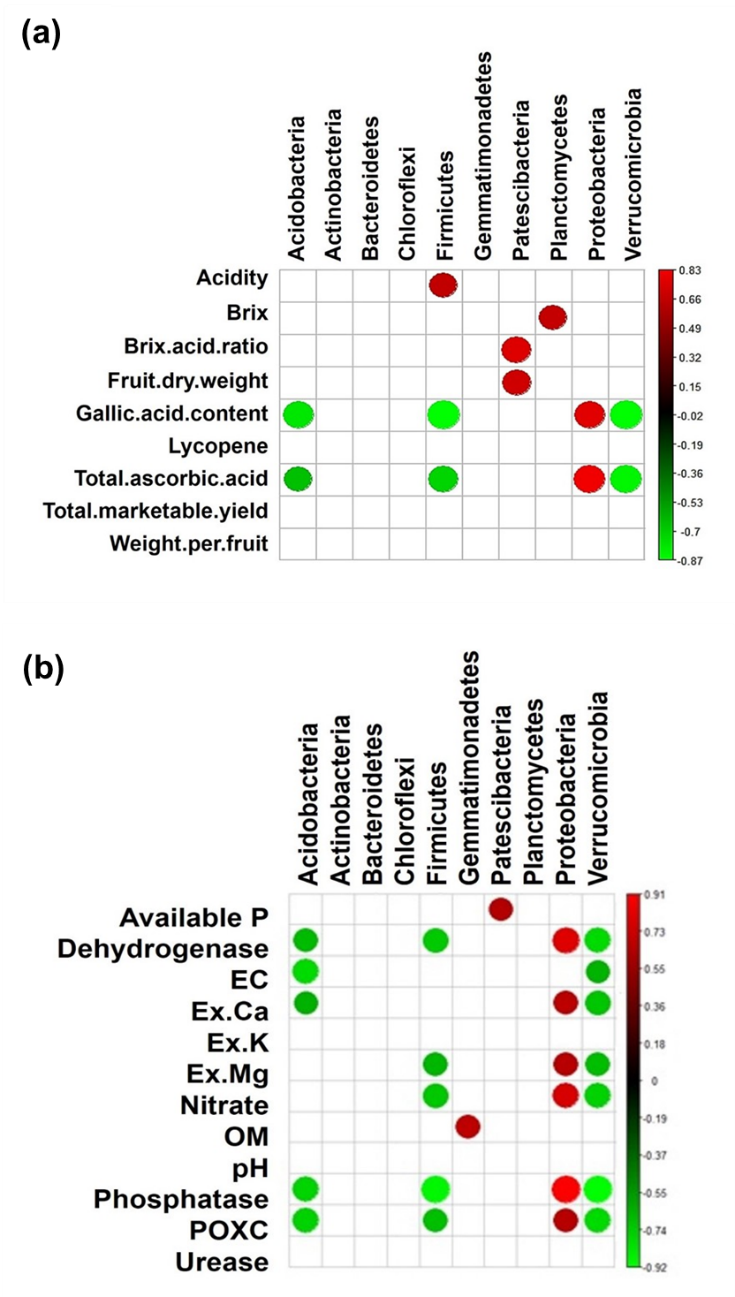


**Fig. S7.** Pearson correlation between the relative abundance of phylum and plant/soil characteristics. (a) Phylum versus tomato harvesting characteristics. (b) Phylum versus soil properties. The red and green nodes represent the positive and negative Pearson’s correlation coefficients (r), considering a *p* value < 0.05, respectively. Each treatment consisted of 5 biological repetitions.

**Table S1.** The properties of the soil in the organic field before treatment were determined to confirm the evenness before inoculation.


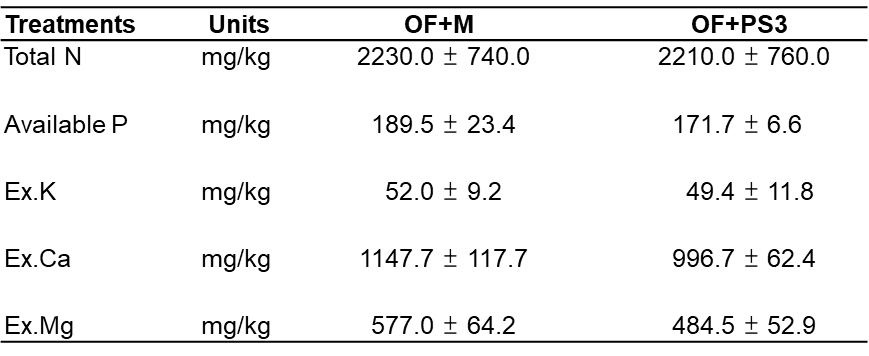


**Table S2.** A description of sequence filtering and chimera removal.


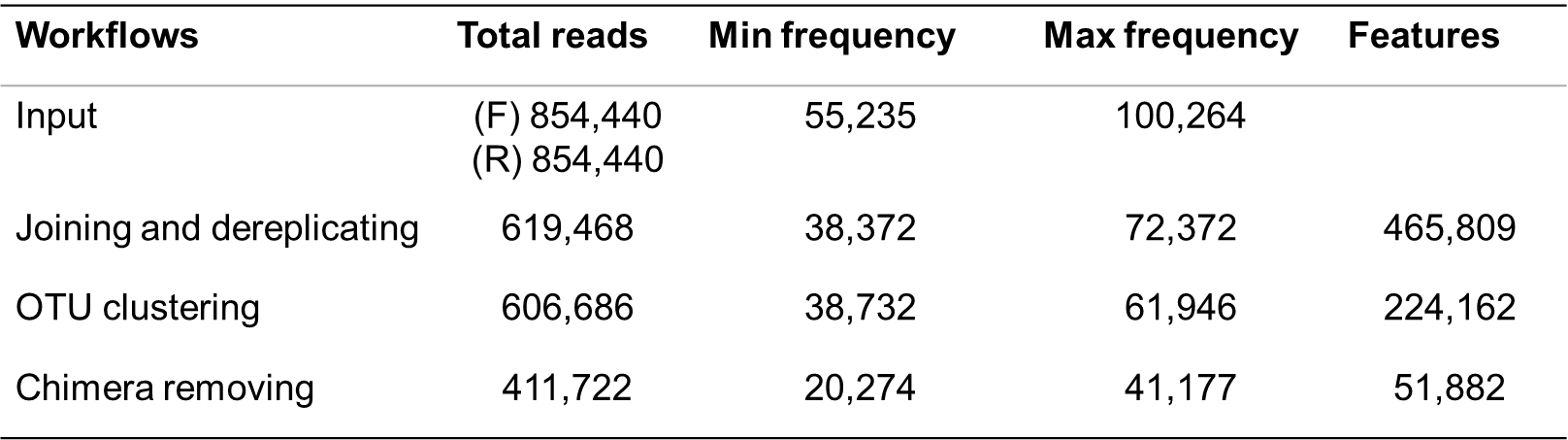


**Table S3.** Primers used for qPCR


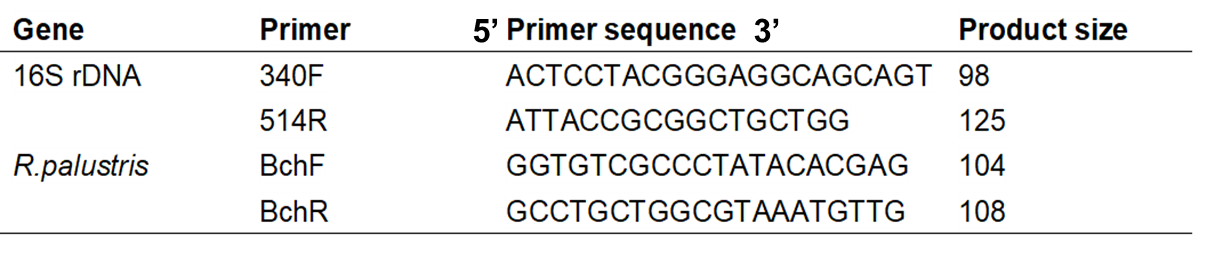


**Table S4.** Summary of weather conditions during the grafted tomato growing season on organic farmland located in Miao Li, Taiwan. This information was obtained from the Central Weather Bureau (CWB).


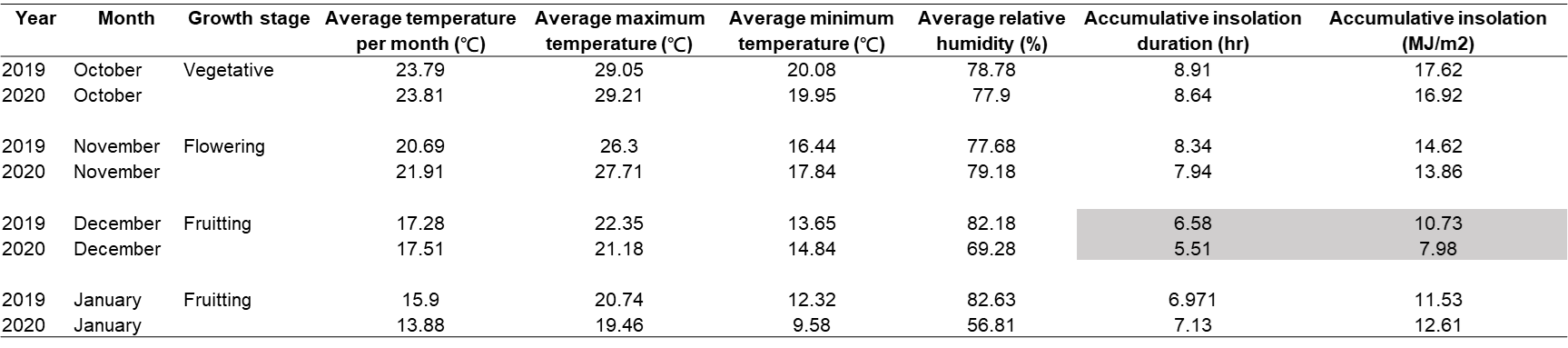

Supplement: Supplementary file 2 [file Table_2.DOCX]
